# Ensemble Machine Learning Models for Predicting Patients With High Usage: Model Validation and Economic Impact Analysis

**DOI:** 10.2196/77202

**Published:** 2026-02-20

**Authors:** Joshua Kuan Tan, Le Quan, Hao Yi Tan, Su-Yen Goh, Julian Thumboo, Marianne Au, Yong Mong Bee, Jen Hong Tan

**Affiliations:** 1 Health Services Research Unit Singapore General Hospital Singapore Singapore; 2 Data Science and Artificial Intelligence Laboratory Singapore General Hospital Singapore Singapore; 3 Future Health Systems Department Singapore General Hospital Singapore Singapore; 4 Group Finance SingHealth Singapore Singapore; 5 Department of Endocrinology Singapore General Hospital Singapore Singapore

**Keywords:** diabetes mellitus, healthcare utilization, population health management, machine learning, artificial intelligence, economic analysis, decision analysis, Monte Carlo simulation

## Abstract

**Background:**

Machine learning models are increasingly used to predict patients at risk of high health care usage for targeted interventions.

**Objective:**

This study aimed to evaluate the predictive performance of multiclass ensemble models across different levels of health care usage and assess their potential application through real-world economic impact analysis.

**Methods:**

A total of 4 previously developed binary classification models (base learners)—boosted trees, multivariate adaptive regression splines, multilayer perceptron, and logistic regression—were extended using a stacking ensemble approach. These base learner models generated individual-level predicted probabilities, which were used as inputs to build multiclass prediction models forecasting usage across defined strata: length of stay (LOS) of <7, 7-13, 14-29, and ≥30 days, and emergency department (ED) visits of <3, 3-4, 5-9, and ≥10 visits. In total, 3 ensemble algorithms were evaluated: random forest, boosted trees, and linear support vector machines. Ensemble models were trained on registry data from 2020-2021 and temporally validated on 2021-2022 data. Performance was assessed using multiclass area under the receiver operating curve, accuracy, and confusion matrix–derived metrics. Economic impact was estimated via Monte Carlo simulations using inpatient billing data, assuming a 20% cost reduction in the following year.

**Results:**

The models were trained on 108,886 patients and validated on 111,004 patients. Among all ensemble configurations, boosted tree models regardless of base learner achieved the highest performance, with multiclass area under the receiver operating curve scores of 0.6877 (95% CI 0.6927-0.7255) for LOS and 0.7601 (95% CI 0.7301-0.7654) for ED visits, and corresponding accuracies of 0.6522 (95% CI 0.6465-0.6579) and 0.7457 (95% CI 0.7405-0.7508), respectively. In the validation set, these models correctly assigned 30.3% of inpatient LOS and 39.8% of ED visits to the correct class, identifying 77% of future inpatient users and 73.9% of future ED users. Economic impact analysis for LOS identified the boosted tree model with logistic regression base learner as dominant, achieving a simulated average cost reduction of SGD $152 million (US $111 million), SGD $2.4 million (US $1.75 million; 1.5%) more than the next best model using a multilayer perceptron base learner.

**Conclusions:**

Ensemble models can effectively predict multilevel health care usage and potentially generate meaningful cost savings when applied to real-world settings. These models may support targeted interventions and guide planning and budgeting in diabetes-related population health programs.

## Introduction

Health care expenditure in high-income countries is rising [1], with chronic diseases (specifically diabetes mellitus) being a leading contributor. Diabetes accounts for approximately 15% of health care expenditure in the United States [2], 9% in the European Union [3], and 11% in Singapore [4]. It is a significant cause of cardiovascular disease, kidney failure, eye disease, peripheral vascular disease, and neuropathy, all associated with substantial morbidity, mortality, and higher health care costs.

Health care spending is also highly skewed, with a small proportion of patients incurring most costs [5]. Efforts to identify high-need, high-cost (HNHC) patients—typically the top 5% of usage or cost—have led to predictive models using regression and machine learning [6]. However, the clinical utility of these models remains uncertain, as few studies assess their net benefit compared with not using them [6-8]. Furthermore, the potential to reduce costs among the top decile or 5% of high-cost patients is smaller than commonly assumed [9], possibly due to heterogeneity in patient characteristics and usage patterns, variability in group membership over time, and a small proportion who are amenable to cost-reducing interventions [5,9,10]. Focusing narrowly on the highest users may also overlook many individuals with future health care needs outside this group.

Building on these insights, our previous study expanded the predictive focus beyond traditional HNHC patients by developing binary classification models to predict inpatient and emergency department (ED) usage across various thresholds for patients in the Singapore Health Services Diabetes Registry (SDR), a clinical database of over 130,000 patients with diabetes [11]. In this study, we extended that work by creating separate multiclass ensemble models for inpatient bed days and ED visits. We validated these models, evaluated their relevance for population health program development, and analyzed their potential economic impact using real-world financial data [7,8]. Our goal is to develop clinically actionable predictive models that can be integrated into broader diabetes-related population health initiatives.

## Methods

### Study Design and Population

This study used data from the SDR spanning 2020-2022, comprising 2 datasets: 2020-2021 and 2021-2022. The 2019-2020 dataset, previously used to develop the binary classification models [11], was excluded from the current analysis to prevent overfitting. Each dataset (2019-2020, 2020-2021, and 2021-2022) was constructed as a cross-sectional snapshot based on a fixed 1-year prediction window. Patients could appear in multiple datasets across years if they continued to meet inclusion criteria for entry into the registry [12]; however, within each dataset, only 1 record per patient per year was used. Multiple visits within the same year were aggregated to derive annual service-level usage metrics (eg, total inpatient bed days and total ED visits).

### Predictor Variables

Individual-level predicted probabilities from the binary classification models, which reflect the predicted likelihood of future usage, were used as predictor variables in the corresponding ensemble models (ie, length of stay [LOS] probabilities for the LOS ensemble model and ED probabilities for the ED ensemble model). Additionally, current-year LOS or ED visits were also included as predictors. Full details on predictor variables for the binary models are provided in Table S1 in Multimedia Appendix 1 and our previous publication [11]; briefly, they include age, gender, ethnicity, housing type, comorbidities (eg, hypertension, hyperlipidemia, and chronic kidney disease), diabetes-related complications, and mean glycated hemoglobin.

### Outcome Variables

Multiclass ensemble models were developed to predict usage for LOS and ED visits across 4 classes each. For LOS, the classes were <7 days, 7-13 days, 14-29 days, and ≥30 days. For ED visits, the classes were <3 visits, 3-4 visits, 5-9 visits, and ≥10 visits.

### Inclusion and Exclusion Criteria

Patients aged 18 years or older with type 2 diabetes mellitus were included. Patients with missing variables were excluded, as data imputation was not performed, and most machine learning algorithms used in this study do not natively support missing values.

### Handling Unbalanced Data

Class imbalance negatively affected model performance in previous studies, and random upsampling outperformed the synthetic minority oversampling technique [11]. We applied random upsampling using the ‘upSample’ function from the *caret* package [13] to achieve equal representation across all classes in the training dataset (ie, an equal number of patients across all classes).

### Machine Learning Models

Previously developed binary classification models from the multi-institutional SDR were used to predict total inpatient LOS and ED visits over a calendar year [11]. Predictions were made across 3 thresholds in the 2 outcomes: LOS of ≥7, ≥14, and ≥30 days; and ED attendance of ≥3, ≥5, and ≥10 visits.

We used the best-performing binary classification models from our previous work, which were trained using the 2019-2020 dataset as base learners: boosted trees, multivariate adaptive regression splines, multilayer perceptron, and logistic regression [11]. For each base learner type, 3 separate binary models were developed to predict inpatient LOS of ≥7, ≥14, and ≥30 days, and ED attendance of ≥3, ≥5, and ≥10 visits, respectively. The individual-level predicted probabilities from these binary models were then used as inputs (meta-features) in a stacking ensemble model designed to classify patients into 4 mutually exclusive outcome categories (LOS: <7, 7-13, 14-29, ≥30 days, and ED: <3, 3-4, 5-9, ≥10 visits).

Stacking was selected over voting-based methods for its ability to learn optimal weights for combining base learner outputs, rather than treating all models equally or assigning manual weights [14]. Furthermore, 3 algorithms were tested as meta-learners: random forest, boosted trees, and linear support vector machines (SVMs). All models were developed in R (version 4.3.1; R Core Team) using the *tidymodels* package [15].

The 2020-2021 dataset was used to train and test the ensemble models, with a 75:25 split. Models were then temporally validated on an unseen 2021-2022 dataset. Each dataset represented a patient‑year observation with a fixed 1‑year prediction horizon. All predictor variables, including inpatient bed days and ED visits, were derived exclusively from the preceding calendar year to predict usage in the subsequent year, ensuring that no future information was used at the time of prediction and avoiding look‑ahead bias. While patients could appear in multiple datasets across years, training and validation were temporally separated to ensure that predictions were evaluated on future, unseen patient‑year data, consistent with real‑world deployment for chronic disease management. To ensure consistency and reproducibility, we used default hyperparameters for all base learners and meta-learners.

### Explainability

We did not conduct explainability analysis for the ensemble models, as previous analyses of the base learner models with model-specific variable importance scores, permutation feature importance plots, and partial dependence plots demonstrated consistent and predictable behavior [11]. The ensemble models’ transparent construction from base learner outputs rendered additional explainability analysis unnecessary.

### Performance Indicators

**Figure 1:**
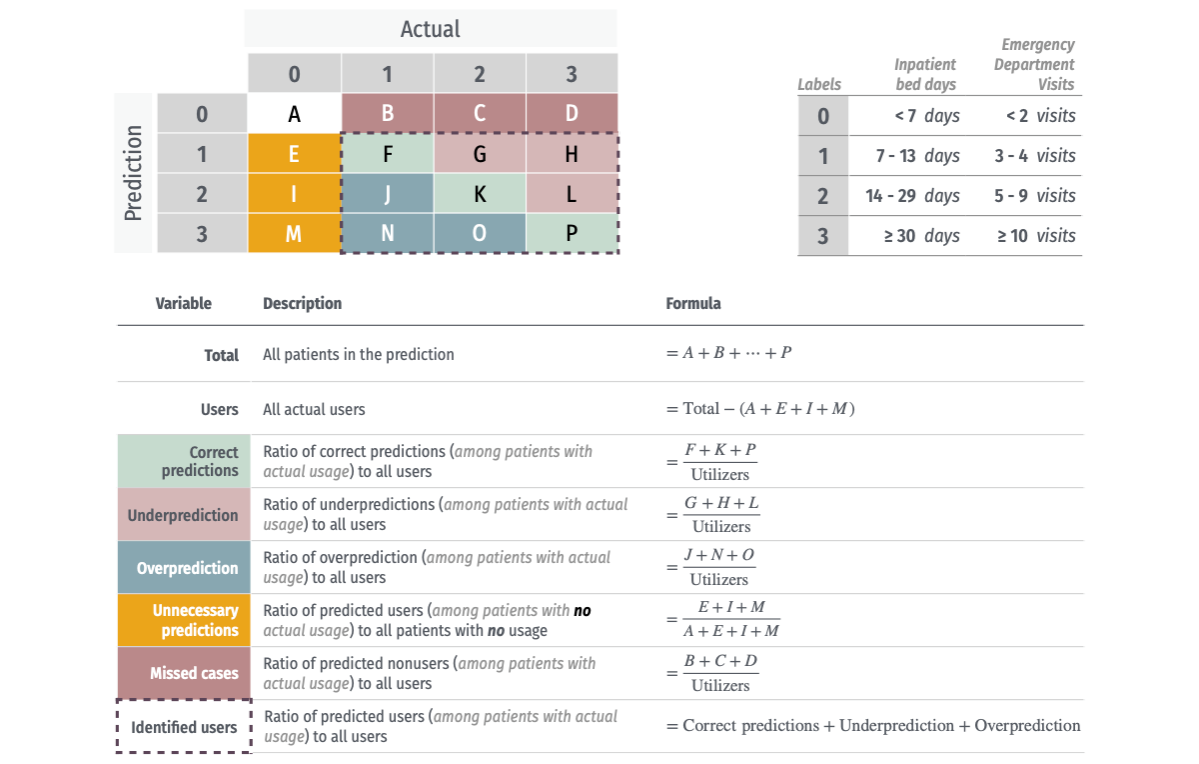
Confusion matrix in a 4-class problem and related evaluation variables.

Model performance was assessed using multiclass area under the receiver operating curve (AUROC; one-vs-rest approach), and accuracy (95% CI) was calculated using the DeLong method (via the pROC package) for AUROC and the confusionMatrix function (from the caret package) for accuracy. Confusion matrices were analyzed, and new metrics specific to our 4-class prediction task were developed for population health programs (Figure 1). Additional metrics, including sensitivity, specificity, positive predictive value, negative predictive value, and balanced accuracy, are detailed in the Multimedia Appendix 1.

These new metrics were designed for population-level interventions using the prediction models. Our priorities were maximizing correct predictions, followed by identifying users, and minimizing unnecessary predictions (ie, patients predicted to have usage but who ultimately did not). Maximizing correct predictions minimizes underpredictions, overpredictions, and missed cases. Increasing identified users enables interventions to reach more patients with future needs, while minimizing unnecessary predictions enhances program efficiency by reducing resources spent on patients unlikely to use services.

### Economic Impact Analysis of the Models

We assessed the potential economic impact of the top 5 ensemble models with the largest proportion of correct predictions for inpatient bed days. Using individual-level inpatient cost data, we modeled hypothetical cost reductions to simulate the financial implications of targeted intervention. These simulations assume that accurately predicted patients would receive effective interventions that reduce future inpatient costs. Cost savings were not attributed to the prediction alone, but to downstream programs triggered by the prediction.

In total, 2 key parameters were incorporated—reach and benefits, both factors important to population-level impact. Reach was defined as the proportion of patients within a predicted usage class who would receive the intervention [16], while benefits referred to the hypothetical percentage reduction in future inpatient costs among those reached, attributable to the intervention.

**Figure 2:**
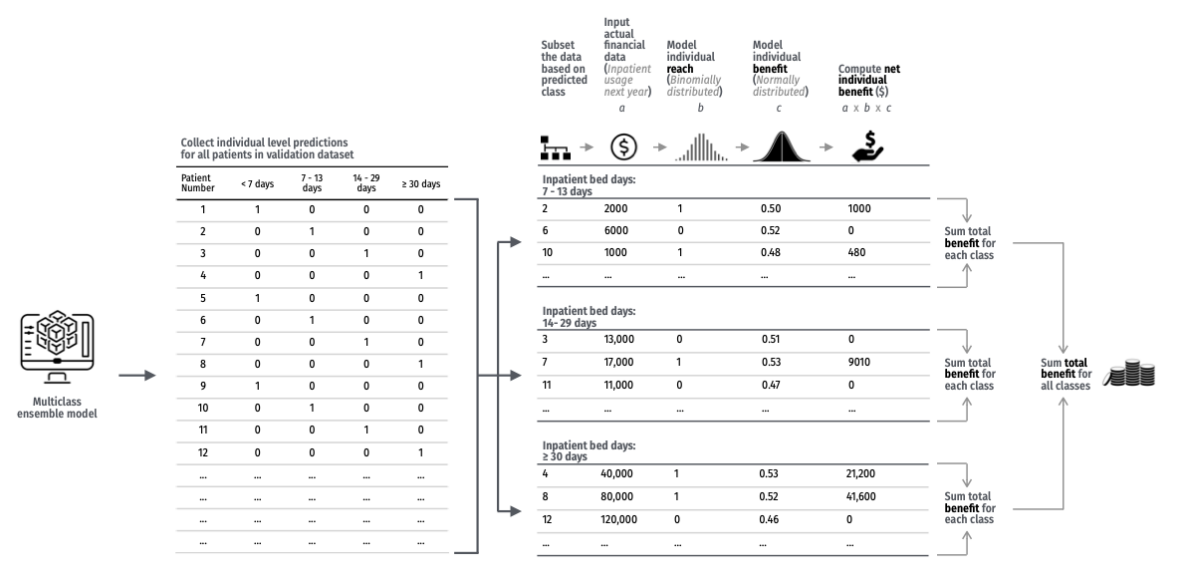
Schematic of economic impact analysis model.

We used total inpatient costs (in Singapore dollars and the equivalent United States dollars [US $], presubsidies) for each calendar year, using the conversion rate of SGD $1=US $1.370, which is accurate as of 1 June 2022. We assessed model performance using fixed effects and Monte Carlo simulations with 10,000 iterations to account for random effects. “Total benefit” was defined as the simulated reduction in inpatient costs in the following year, enabling us to compare models and identify the dominant one. A schematic of the economic impact analysis model is provided in Figure 2, with parameters detailed in Table S2 in Multimedia Appendix 1.

In the fixed effects model, we modeled the benefit at a uniform 50% cost reduction for all identified patients—an optimistic scenario to benchmark maximum potential impact. In contrast, the Monte Carlo simulations modeled a more conservative and realistic benefit at an average cost reduction of 20% across all classes, with stochastic variation applied using a normal distribution.

Real-world programmatic use was further simulated by varying reach across usage tiers: average reach levels were set at 90% for the highest usage class, 70% for the middle class, and 50% for the lowest classes, each with a 5% variance. For example, the reach for the highest users ranged from 85% to 95% across simulations. This tiered approach reflects potential operational realities: patients predicted in the highest usage class are fewer in number and more likely to be prioritized and successfully engaged by targeted interventions. In contrast, lower usage classes include larger populations, making it less feasible to reach all patients in practice. To account for individual variability in intervention uptake, reach levels were also modeled using a binomial probability distribution.

From the Monte Carlo simulations, we identified the dominant model as the one with the highest median cost savings. To explore uncertainty and assess the robustness of this selection, we performed parameter sensitivity analysis stratified by usage class. Specifically, we varied the reach and benefit parameters in 10% intervals (ranging from 10% to 90%), resulting in 81 unique parameter combinations. For each combination, 1000 Monte Carlo runs were conducted to account for stochastic variability. The distribution of cost savings across simulations was analyzed, and results were visualized using the *plotly* package [17] to provide an interactive and detailed view of model performance under different programmatic scenarios.

### Reporting Checklist

We reported our study using the Transparent Reporting of a multivariable prediction model for Individual Prognosis or Diagnosis + Artificial Intelligence (TRIPOD+AI) checklist (Multimedia Appendix 2) [18].

### Ethical Considerations

Ethics approval was obtained from the Singapore Health Services Centralized Institutional Review Board before initiating this study (reference 2022/2133). As all participant data were deidentified, a waiver for participant consent was also obtained. All procedures were performed in compliance with relevant laws and institutional guidelines.

## Results

### Characteristics of the Datasets

The demographics, comorbidities, and usage characteristics of the datasets have been previously described [11]. The 2020-2021 and 2021-2022 datasets included 108,886 of 140,859 (77.3%) patients and 111,004 of 137,584 (80.7%) patients from the total SDR cohort, respectively. Total inpatient costs (presubsidies) per annum are summarized in Table 1, and inpatient LOS distributions are illustrated in Figures S1-S3 in Multimedia Appendix 1.

Inpatient usage showed an increasing trend, with gross inpatient bills (unadjusted for inflation) rising from a nominal value of SGD $808 (US $590) million in 2019 to SGD $1.01 billion (US $737 million) in 2022. The proportion of patients with inpatient usage increased from 46,686 of 134,670 (34.7%) in 2019 to 51,228 of 137,627 (37.2%) in 2022, while the average cost per patient rose from SGD $17,321 to SGD $19,704 (US $12,643 to US $14,382). Total inpatient costs were heavily right-skewed (Table S3 in Multimedia Appendix 1) and followed a Pareto distribution (Figure S1 in Multimedia Appendix 1), with the top 10% of patients accounting for 80% of cumulative health care costs and inpatient bed days annually (Figures S1 and S2 in Multimedia Appendix 1). A strong correlation was observed between gross inpatient bills and inpatient bed days (Figure S3 in Multimedia Appendix 1).

**Table 1:** Performance metrics of the top 5 best-performing ensemble models.

Model characteristics				Accuracy (95% CI)		Multiclass AUROC^a^ (95% CI)		Correct predictions, %		Underpredictions, %		Overpredictions, %		Unnecessary predictions, %		Identified users, %		Missed cases, %
**Best performing ensemble models on test dataset (2021-2022)**																		
	**Predicting inpatient bed days category**																	
		Boosted tree with MARS^b^ as base learner	0.6522 (0.6465-0.6579)		0.6877 (0.6927-0.7255)		30.5		23.2		24.1		31.3		77.8		22.2	
		Boosted tree with MLP^c^ as base learner	0.6685 (0.6629-0.6741)		0.6832 (0.6875-0.7202)		30.2		20.3		25.5		29.6		76		24	
		Boosted tree with logistic regression as base learner	0.6495 (0.6438-0.6552)		0.6696 (0.6896-0.722)		30.2		20		26		31.7		76.1		23.9	
		Boosted tree with boosted tree as base learner	0.6654 (0.6598-0.671)		0.6776 (0.6936-0.7259)		29.7		21.2		24.1		29.9		75.1		24.9	
		Linear SVM^d^ with MLP as base learner	0.7300 (0.7246-0.7352)		0.6758 (0.6757-0.7089)		26.8		19.7		22.6		22.6		69.1		30.9	
	**Predicting emergency department visits category**																	
		Boosted tree with MARS as base learner	0.7457 (0.7405-0.7508)		0.7601 (0.7301-0.7654)		39.5		10.7		24.8		24		75		25	
		Boosted tree with logistic regression as base learner	0.7205 (0.7151-0.7258)		0.7569 (0.7294-0.7641)		39.3		11		24.9		26.6		75.2		24.8	
		Boosted tree with MLP as base learner	0.7432 (0.738-0.7484)		0.7557 (0.731-0.7664)		37.9		10.4		25.8		24.2		74		26	
		Boosted tree with Boosted tree as base learner	0.7425 (0.7372-0.7476)		0.7527 (0.7016-0.7378)		37.1		14.7		18.3		24.2		70.1		29.9	
		Linear SVM using MARS as base learner	0.7849 (0.7800-0.7898)		0.7722 (0.7316-0.7681)		30.3		6.6		34.8		19.6		71.8		28.2	
**Best performing ensemble models on validation datasets**																		
	**Predicting inpatient bed days category**																	
		Boosted tree with MARS as base learner	0.6405 (0.6377-0.6434)		0.6811 (0.6986-0.714)		30.3		21.1		25.6		32.1		77		23	
		Boosted tree with boosted tree as base learner	0.6496 (0.6468-0.6524)		0.6828 (0.6966-0.712)		29.9		21.9		24.7		31.1		76.4		23.6	
		Boosted tree with logistic regression as base learner	0.6357 (0.6329-0.6385)		0.6767 (0.6929-0.7082)		29.4		20.2		27.6		32.6		77.1		22.9	
		Boosted tree with MLP as base learner	0.6558 (0.6530-0.6586)		0.6782 (0.6957-0.7111)		29.2		19.2		27.4		30.3		75.8		24.2	
		Linear SVM with MLP as base learner	0.71 (0.7073-0.7127)		0.6742 (0.6851-0.7004)		26.5		19.3		24.5		24		70.4		29.6	
	**Predicting emergency department visits category**																	
		Boosted tree with logistic regression as base learner	0.7053 (0.7026-0.7079)		0.7669 (0.7199-0.7359)		39.8		10.4		23.7		27.9		73.9		26.1	
		Boosted tree with boosted tree as base learner	0.7317 (0.7290-0.7343)		0.7577 (0.7139-0.73)		39.7		12.5		19		25.1		71.1		28.9	
		Boosted tree with MLP as base learner	0.7269 (0.7243-0.7295)		0.7563 (0.7158-0.732)		36.9		10.5		23.9		25.5		71.3		28.7	
		Boosted tree using MARS as base learner	0.7266 (0.7239-0.7292)		0.7664 (0.7169-0.7332)		36.7		10.2		24.7		25.5		71.5		28.5	
		Linear SVM using MARS as base learner	0.7656 (0.7631-0.7681)		0.7818 (0.7204-0.7368)		30.1		6.7		32.2		21		69		31	

^a^AUROC: area under the receiver operating curve.

^b^MARS: multivariate adaptive regression splines.

^c^MLP: multilayer perceptron.

^d^SVM: support vector machine.

### Multiclass Model Performance

We developed 12 ensemble models by combining 4 base learner models with 3 ensemble algorithms: random forest, boosted trees, and linear SVM. Detailed performance metrics for all 12 models on the test dataset are provided in Table S4-S6 in Multimedia Appendix 1. We then selected top 5 models for each prediction task based on the highest percentages of correct predictions and identified users (Table 1 and Table S4 in Multimedia Appendix 1), and their performance was temporally validated on the unseen dataset from 2021 to 2022; detailed performance metrics for these models on the test dataset are provided in Tables S7 and S8 in Multimedia Appendix 1.

Boosted tree models outperformed other ensemble algorithms across metrics, including multiclass AUROC, correct predictions, and identified users. While linear SVM models had comparable multiclass AUROC scores to boosted tree models, they showed significantly lower correct predictions, fewer identified users, and more missed cases. Random forest models performed poorly across all metrics.

Boosted tree models demonstrated consistent performance on test and validation datasets, indicating robustness on unseen data (Table 1 and Table S4 in Multimedia Appendix 1). For inpatient bed days, these models correctly assigned approximately 30% of future users to the appropriate category, identified >75% of future users, and generated 30%-32% unnecessary predictions among nonusers. For ED visits, they correctly assigned 36%-39% of future users, identified 70%-75% of users, and generated approximately 25% unnecessary predictions among nonusers.

### Economic Impact Analysis of the Models

Results from the fixed effects and Monte Carlo simulations are presented in Table 2 and Figure S4 in Multimedia Appendix 1. The boosted tree ensemble model with logistic regression as the base learner emerged as the dominant model, although other boosted tree models performed similarly. In the fixed effects simulation, the dominant model generated an estimated benefit of SGD $210 (US $153) million. In the Monte Carlo simulations, it produced a median benefit of SGD $152 (US $110) million. Notably, nearly half of the simulations for the dominant model exceeded the 75th percentile of simulations from other models (Table 2 and Figure S4 in Multimedia Appendix 1).

**Table 2:** Results of fixed effects and Monte Carlo simulations.

Variables			Models								
			Boosted tree with logistic regression as base learner		Boosted tree with MLP^a^ as base learner		Boosted tree with MARS^b^ as base learner		Boosted tree with boosted tree as base learner		Linear SVM^c^ with MLP as base learner
**Results for the fixed effects simulation (SGD $)**											
	Total simulated reduction in inpatient bill	210,220,829		205,184,563		209,535,576		206,000,929		189,308,087	
**Results for the Monte Carlo simulations (SGD $)**											
	Mean (SD)	152,794,862 (6,001,058)		150,437,894 (5,816,301)		149,408,731 (5,959,678)		147,699,501 (5,809,931)		136,692,927 (7,365,761)	
	Median (IQR)	152,854,645 (8,656,193)		150,468,563 (8,399,851)		149,405,404 (8,402,405)		147,756,137 (8,198,816)		136,722,177 (12,202,993)	
	Range (minimum-maximum)	134,202,838-170,594,658		133,162,981-167,868,627		131,679,740-167,051,047		129,415,870-165,742,786		118,420,401-153,678,680	
	Q25	148,510,936		146,256,897		145,189,862		143,596,095		130,577,413	
	Q75	157,167,129		154,656,748		153,592,267		151,794,911		142,780,406	

^a^MLP: multilayer perceptron.

^b^MARS: multivariate adaptive regression splines.

^c^SVM: support vector machine.

### Parameter Sensitivity Analysis

**Figure 3:**
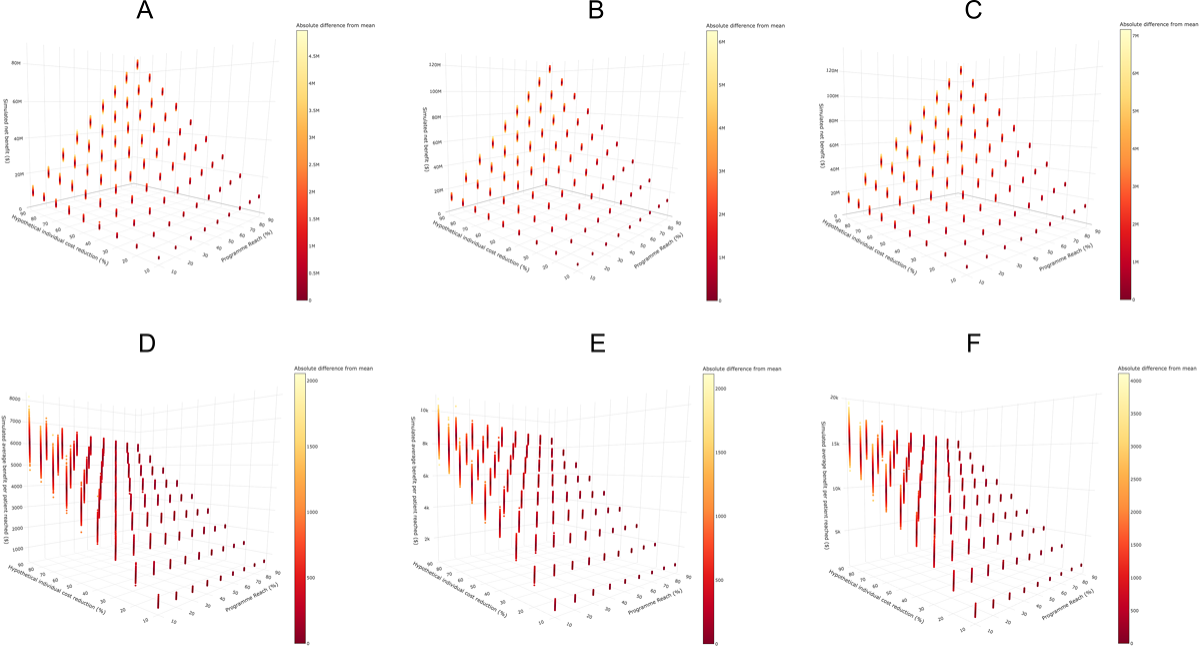
3D scatterplot for Monte Carlo simulations across various permutations of reach and benefit for the boosted tree with logistic regression as base learner ensemble. (A) Simulated net benefit for prediction 7-13 days; (B) simulated net benefit for prediction 14-29 days; (C) simulated net benefit for prediction ≥30 days; (D) simulated average benefit per patient for prediction 7-13 days; (E) simulated average benefit per patient for prediction 14-29 days; and (F) simulated average benefit per patient for prediction ≥30 days. Readers can refer to the interactive plots on GitHub for higher resolution interactive images.

We evaluated the dominant model’s performance across various permutations of reach and benefit (Figure 3 and interactive plots available on GitHub [19]). As expected, the net benefit increased with higher individual cost reductions (Figures 3A-3C) and program reach. For predicting LOS of 14-29 days (Figure 3B), with a constant 50% cost reduction, the simulated net benefit ranged from SGD $5.95 to SGD $9.62 (US $4.34 to US $7.02) million at 10% reach, SGD $35.6 to SGD $40.7 (US $26.0 to US $29.7) million at 50% reach, and SGD $66.6 to SGD $70.0 (US $48.6 to US $51.1) million at 90% reach.

With increasing program reach, the range of simulated average benefit per patient narrowed. For LOS of 14-29 days (Figure 3D), with a 50% cost reduction, the average benefit per patient ranged from SGD $3708 to SGD $5814 (US $2707 to US $4244) at 10% reach and from SGD $4656 to SGD $4389 (US $3399 to US $3204) at 90% reach. The implications of these findings are further discussed in the Discussion section.

## Discussion

### Principal Findings

In this study, we expanded on our previous work by developing multiclass ensemble machine learning models to predict future inpatient LOS and ED visits. The models were evaluated using public health program objectives, analyzing predictions through confusion matrices and performance metrics to identify the best-performing models. Economic impact analysis, incorporating inpatient cost data and program-related simulations, identified the boosted tree ensemble algorithm with logistic regression as the base learner as dominant for LOS. Further testing across analytical parameters confirmed its robustness and practical applicability.

While previous studies focused on binary outcomes with limited evaluation of clinical utility [6,20], our study reframes high health care usage as a multiclass problem, enabling more nuanced predictions across different health care events. By incorporating financial data into the economic impact analysis with Monte Carlo simulations, we model the potential benefits of the model, enhancing its relevance for real-world applications [7,8]. This novel approach to economic impact analysis represents a significant contribution to machine learning in health care and the design of resource-efficient health programs.

### Advancing High User Prediction Through Multiclass Modeling and Economic Impact Analysis

Our study advances the prediction and targeting of patients with high health care usage by moving beyond binary classification. While many previous studies have focused on predicting binary outcomes—such as identifying HNHC patients in the top 5% or 10% of usage—this strategy has limited practical utility, as most high users do not remain so over time [9,21,22]. Consequently, there is growing recognition of the need to predict risk across multiple levels of usage, enabling stratified interventions tailored to predicted resource needs [9].

To the best of our knowledge, few studies have applied machine learning to predict health care usage across multiple discrete classes. One recent study using Louisiana Department of Health records built multinomial models to predict clinical service usage (ie, number of clinic visits across 5 classes), and found that tree-based ensemble methods, such as extreme gradient boosting and random forest, consistently outperformed logistic regression, neural networks, and naïve Bayes models—likely due to their capacity to model complex nonlinear interactions in heterogeneous data [23]. Another study within the New York City safety net system developed multiple models to predict acute care usage as both binary and continuous outcomes. Their best-performing model, which predicted the number of acute care days as a continuous variable, successfully identified approximately half of the patients who would go on to have very high acute care use [24].

Our study builds on these approaches in several novel ways. First, we applied a stacking ensemble framework to a multiclass prediction problem, enabling stratification across 4 tiers of inpatient bed days and ED visits. This allows for more granular predictions and targeted interventions, unlike previous work focused solely on binary or continuous outcomes. Second, we introduced custom evaluation metrics derived from confusion matrices (Figure 1) that are directly relevant to population health program planning. Third, we conducted a Monte Carlo–based economic impact analysis, incorporating real-world inpatient cost data to estimate potential cost savings under varying assumptions of reach and benefit. This coupling of predictive modeling with economic simulation offers a practical and policy-relevant contribution to the literature on machine learning for health care resource optimization.

### Practical Usage of Ensemble Prediction Models for Future Inpatient Bed Days and ED Visits

Our study predicted LOS and ED usage across 4 tiers, rather than focusing on a specific percentile as is the case in many previous studies [6]. This approach enables tailored interventions based on predicted resource use. The distribution of predictions in the confusion matrix (Tables S4 and S5 in Multimedia Appendix 1), with more predictions in the lower usage tier and fewer in the highest usage tier, suggests that low-cost, scalable interventions (eg, clinical flags, EMR risk prompts, or light-touch monitoring) may be suitable for the lower tier (7-13 inpatient days or 3-4 ED visits) [9,10]. For the middle tier (14-29 inpatient days or 5-9 ED visits), moderately scalable interventions, such as programs involving community nurses or care coordinators, may be effective. Higher-cost, intensive interventions are needed for the highest tier (≥30 inpatient days or ≥10 ED visits), although the scalability is limited.

The new model evaluation metrics we developed address the varying costs of misclassification [25]. Models achieving high correct predictions and low unnecessary predictions enhance program efficiency, while those with more identified users allow interventions to reach more patients, although subsequent recalibration of the intervention may be required downstream. Future health programs using these models should assess patient characteristics and clinical needs at enrollment and during follow-up to ensure appropriate intervention assignment. These metrics may also be adapted for other multiclass problems in health care.

Balancing trade-offs between correct and unnecessary predictions is crucial when selecting the best model. Models with higher correct predictions often had more unnecessary predictions (Tables S4-S6 in Multimedia Appendix 1). Similarly, models with more identified users showed higher unnecessary predictions. This trade-off highlights the need to balance these metrics. Although unnecessary predictions may reduce program efficiency, prioritizing correct predictions and identified users led to greater economic benefits, as shown in our economic impact analyses (Table 2 and Figure S4 in Multimedia Appendix 1). Boosted tree ensemble models, which had higher correct predictions, identified users and unnecessary predictions, and significantly outperformed linear SVM ensemble models.

An important feature of our ensemble models is the tiered distribution of predictions (Tables S4-S7 in Multimedia Appendix 1), with the fewest in the highest tier, followed by the middle, and most in the lower tier. This distribution supports efficient resource allocation: low-cost, scalable interventions can be directed to lower tiers, while high-cost, intensive interventions are reserved for the highest tier—ensuring sustainable deployment of resources.

This stratified output also enables a graduated intervention pathway, starting with risk-appropriate outreach and followed by clinical reassessment to adjust intervention intensity. This flexibility is essential given the modest positive predictive value (13%-14%), even in the highest usage group—a reflection of the complexity and unpredictability of health care use, driven by diverse clinical and social factors.

Nevertheless, our best models identified more than 75% of future high users (≥7 inpatient days), offering valuable opportunities for early engagement. Even if further triage is needed, this early identification is a key step in proactive population health management. Future implementations should incorporate qualitative review or clinical vetting to further optimize targeting and minimize risks of overintervention or alert fatigue.

### Implications of Economic Impact Analysis and Parameter Sensitivity Analysis

To our knowledge, this is the first study to conduct an economic impact analysis of predictive models for identifying patients at risk of high health care usage. We selected the best-performing ensemble model for inpatient LOS, leveraging inpatient billing data to assess its real-world potential. The boosted tree ensemble algorithm with logistic regression as its base learner emerged as the dominant model (Table 2 and Figure 3), demonstrating superior performance in both fixed effects and Monte Carlo simulations. Notably, the median simulation outcome for the dominant model exceeded the 75th percentile of other models, indicating that its typical performance surpassed even the best-case scenarios (upper quartile) of the alternatives. This highlights the dominant model’s consistency and reliability, despite similar AUROC, accuracy, and evaluation metrics across models.

Findings from the parameter sensitivity analysis provide health care planners with insights into the potential cost savings on different combinations of reach and benefit. Our results show that increasing program reach significantly boosts total benefit, while reducing variability in benefit per patient, especially when more high-cost patients (typically those found in the top cost percentiles) are included. This reflects the Pareto distribution of inpatient costs (Figure S1 in Multimedia Appendix 1), where maximizing program reach is essential for capturing high-cost patients and maximizing the overall net benefit.

While the parameter sensitivity analysis does not explicitly incorporate intervention costs, it serves as a useful planning tool by simulating the financial space available for intervention. For example, in a conservative scenario targeting patients predicted to stay 14-29 days, assuming a 30% cost reduction and 50% reach, projected savings reach SGD $21.0 (US $15.3) million in a year. This estimate can guide budget planning, allowing programs to allocate resources up to this amount while maintaining a positive return on investment. Future iterations of this analysis could incorporate cost parameters and extend to multiyear projections, enhancing its utility for implementation planning.

### Limitations

We acknowledge the potential for selection bias arising from the exclusion of patients with missing variables. Complete case analysis was used because data imputation was not performed, and several of the machine learning algorithms applied in this study do not natively support missing values. Nevertheless, a substantial proportion of the registry was retained: 74.6% (100,500/134,670) of eligible patients were included in training the binary base learners, 77.3% (108,886/140,859) in training the stacking ensemble models, and 80.7% (111,004/137,584) in the validation dataset as detailed in our previous study [11]. Patients excluded due to missing data were more likely to have limited interaction with the health care system (eg, no inpatient, ED, or primary care visits), and their exclusion is unlikely to materially affect model performance for predicting higher levels of usage. However, we acknowledge that missingness may be informative, and future work could explore multiple imputation strategies or algorithms that explicitly handle missing data to further assess the robustness and generalizability of the findings.

A methodological limitation is the potential for logical inconsistencies in the predicted probabilities generated by independently trained binary classifiers used as inputs to the stacking ensemble model. For instance, a patient may receive a higher predicted probability for LOS≥14 days than for LOS≥7 days, which violates logical ordering. While these probabilities were not used directly to assign final classes, serving instead as features for a stacking ensemble meta-learner, such inconsistencies could still affect interpretability and calibration. We did not formally assess the prevalence of these inconsistencies in this study. However, we acknowledge this limitation and plan to explore ordinal-consistent modeling strategies in future work, such as classifier chains or ordinal regression frameworks that preserve monotonicity across outcome thresholds.

Additionally, we did not benchmark against simpler baselines, such as heuristic rules (eg, previous year usage) or native multiclass algorithms. Future work could explore these comparisons to better understand the incremental benefits of ensemble architectures in terms of complexity, predictive performance, and operational interpretability.

Another methodological consideration is the absence of hyperparameter tuning, which may have improved absolute model performance. Our objective in this study was to evaluate relative model performance across ensemble strategies and to develop a transparent and reproducible framework. Default hyperparameters were used to ensure consistency and comparability. We acknowledge that in real-world deployment, targeted hyperparameter tuning would likely be performed to further optimize predictive accuracy, and we identify this as a direction for future work.

Relatedly, we did not perform standard decision curve analysis as our multiclass models output discrete class labels corresponding to stratified intervention tiers rather than risk probabilities used for binary decisions. Future work could explore ordinal or multiclass extensions of decision curve analysis that may better reflect stratified care models and potentially enhance decision support performance.

Additionally, in our economic simulations, we also assumed normally distributed variation in intervention benefit; while this was suitable for benchmarking, we acknowledge that future work could explore log-normal or gamma distributions.

A further consideration is the potential impact of pandemic-related disruptions during the study period, which coincided entirely with the COVID-19 pandemic (2020-2022). As the ensemble models were trained and validated on temporally distinct datasets from this period, unmeasured shifts in disease burden and health care delivery—such as deferred elective care, changes in admission criteria, infection control protocols, ED triage processes, and inpatient discharge practices—may have influenced LOS and ED usage patterns. While a detailed exploration of these pandemic-related dynamics is beyond the scope of this study, we acknowledge that they may have affected model performance and limited generalizability to nonpandemic settings. We intend to validate the models using postpandemic data (eg, 2024 onwards) in future work to assess robustness under more stable health care conditions.

Another important limitation is the real-world variability in health care usage among high users; the best models could correctly assign only about 30% of future users to the appropriate category. This variability, likely influenced by regression to the mean and the inherent complexity of the prediction task, highlights the need for additional program measures, such as downstream assessments of predicted patients.

From a pragmatic perspective, not all high users may respond to interventions, and real-world efficacy is likely to vary depending on clinical or social drivers [6]. Our economic impact analyses assumed average cost reductions across usage tiers to facilitate comparative evaluation of ensemble models, but we acknowledge this simplification may not capture the full heterogeneity of intervention effects. Future work could explore stratified modeling based on clinical or social risk profiles when such data becomes available. However, by predicting varying levels of resource use, our models allow for interventions to target patients who may be more amenable to them [9]. This remains an area requiring further research to pinpoint patients with modifiable trajectories [26]. It has been suggested that prediction models should combine quantitative prediction modeling with qualitative assessment for better case identification [6]. In line with this, we plan to explore the use of large language models to analyze unstructured clinical text and better understand social determinants of health [27], supporting a more tailored and prescriptive approach to patient management.

### Conclusions

We used binary classification machine learning algorithms to develop ensemble models predicting future health care usage, focusing on inpatient LOS and ED visits. These models were evaluated using metrics relevant to population health programs. Economic and parameter sensitivity analyses of the best-performing models offered valuable insights for designing effective health initiatives. Our ensemble model could be integrated into a diverse decision-making framework, providing tailored recommendations for patients with predicted high health care usage.

## Appendix

Multimedia Appendix 1 Additional tables and figures.

Multimedia Appendix 2 TRIPOD+AI checklist.

## Abbreviations

AUROC: area under the receiver operating characteristic curve
ED: emergency department
HNHC: high-need, high-cost
LOS: length of stay
SDR: Singapore Health Services Diabetes Registry
SVM: support vector machine
TRIPOD+AI: Transparent Reporting of a multivariable prediction model for Individual Prognosis or Diagnosis + Artificial Intelligence

## Data Availability

The datasets generated and/or analyzed during this study are not publicly available due to Singapore Health Services Cluster Data Policy on data sharing restrictions. Data are available from the Singapore Health Services Diabetes Registry Disease Registry Committee for researchers who meet the criteria for access to confidential data. The criteria include institutional review board approval, data use approval, and a research collaboration agreement. The point of contact for the Singapore Health Services Diabetes Registry Disease Registry Committee is Lee Thong Shuen (email: lee.thong.shuen@singhealth.com.sg).

The underlying code for this study is not publicly available for proprietary reasons but may be made available to qualified researchers on reasonable request from the corresponding author.
