# Supplementary material for: Ensemble Machine Learning Models for Predicting Patients With High Usage: Model Validation and Economic Impact Analysis
Source: JMIR Med Inform. 2026 Feb 20;14:e77202. doi: 10.2196/77202 (PMC12966819; doi:10.2196/77202)

**Multimedia Appendix 1**

**Contents**

**Table S1.** Variables utilized in binary classification base learner models.
**Table S2.** Parameters for economic impact analyses.

**Table S3.**  Characteristics and distribution of unadjusted gross inpatient bill (per calendar year) for the SDR.

**Table S4.** Performance of ensemble models on test dataset.

**Table S5**. Detailed performance metrics for all models predicting inpatient bed days (test dataset).

**Table S6**. Detailed performance metrics for all models predicting emergency department visits (test dataset).

**Table S7**. Detailed performance metrics for top 5 selected models predicting inpatient bed days (validation dataset).

**Table S8**. Detailed performance metrics for top 5 selected models predicting emergency department visits (validation dataset).

**Figure S1**. Two-axis plot for the distribution of gross inpatient bill (per calendar year) in the SDR.

**Figure S2.** Two-axis plot for the distribution of total inpatient bed days in the SDR*.*

**Figure S3.** Scatterplots demonstrating the relationship between gross inpatient bill and inpatient bed days.

**Figure S4**. Boxplot for Monte Carlo simulations

**Table S1. Variables utilized in binary classification base learner models.**

| No. | Variables | Description of variable |
| --- | --- | --- |
| 1 | Age | Continuous |
| 2 | Gender | Dichotomous (Male, female) |
| 3 | Ethnicity | Categorical (Chinese, Malay, Indian, other) |
| 4 | Housing type | Categorical (One and two-room flat, three-room flat, four-room flat, five-room flat, private condominium, private landed property) |
| 5 | Rental block | Dichotomous (Absent, present) |
| 6 | Singapore Housing Index | Continuous |
| 7 | Hypertension | Dichotomous (Absent, present) |
| 8 | Hyperlipidemia | Dichotomous (Absent, present) |
| 9 | Mean Hba1c | Continuous |
| 10 | DM meds category | Categorical (None, oral only, insulin only, oral and insulin) |
| 11 | CKD stage | Categorical (Stage 1, stage 2, stage 3A, stage 3B, stage 4, stage 5) |
| 12 | Dialysis | Dichotomous (Absent, present) |
| 13 | IHD | Dichotomous (Absent, present) |
| 14 | Peripheral arterial disease | Dichotomous (Absent, present) |
| 15 | Stroke (hemorrhagic) | Dichotomous (Absent, present) |
| 16 | Stroke (ischemic) | Dichotomous (Absent, present) |
| 17 | Major lower extremity amputation (LEA) | Dichotomous (Absent, present) |
| 18 | Minor lower extremity amputation (LEA) | Dichotomous (Absent, present) |
| 19 | Diabetic foot | Dichotomous (Absent, present) |
| 20 | Diabetic eye complications | Dichotomous (Absent, present) |
| 21 | Nephropathy | Dichotomous (Absent, present) |
| 22 | Neuropathy | Dichotomous (Absent, present) |
| 23 | Present year inpatient bed days | Categorical (0 days, 1-2 days, 3-6 days, 7-13 days, 14-29 days, ≥ 30 days) |
| 24 | Present year number of emergency department visits | Interval |

See manuscript for details: Tan JK, Quan L, Salim NNM, Tan JH, Goh SY, Thumboo J, Bee YM. Machine Learning-Based Prediction for High Health Care Utilizers by Using a Multi-Institutional Diabetes Registry: Model Training and Evaluation. JMIR AI. 2024 Oct 17;3:e58463. doi: 10.2196/58463. PMID: 39418089; PMCID: PMC11528163.

**Table S2**. Parameters for economic impact analyses.

| **Fixed effects model** | **Monte Carlo Simulation of random effects model** |
| --- | --- |
| Reach not modelled | Reach 3_mean_ : 90%  Reach 3_max_ : 95%  Reach 3_min_ : 85%  Reach 2_mean_ : 70%  Reach 2_max_ : 80%  Reach 2_min_ : 60%  Reach 1_mean_ : 50%  Reach 1_max_ : 60%  Reach 1_min_ : 40%    Benefit 3_mean_: 20% reduction in cost  Benefit 2_mean_: 20% reduction in cost  Benefit 1_mean_: 20% reduction in cost  SD_benefit_ 3: 0.05  SD_benefit_ 2: 0.05  SD_benefit_ 1: 0.05 |
| Benefit 3: 50% reduction in cost  Benefit 2: 50% reduction in cost  Benefit 1: 50% reduction in cost |  |

**Table S3.**  Characteristics and distribution of unadjusted gross inpatient bill (per calendar year) for the SDR.

| Registry & Financial year | Distribution of costs (S$) | | | | | | Total cost of gross inpatient bill in the SDR (S$) | Registry size (n) | No. of patients with inpatient utilization (%) | Average cost per patient with inpatient utilization (S$) |
| --- | --- | --- | --- | --- | --- | --- | --- | --- | --- | --- |
|  | Min. | Q1 | Median | Mean | Q3 | Max. |  |  |  |  |
| 2019 | $0 | $0 | $0 | $6,005 | $3,324 | $953,460 | $808,639,638 | 134,670 | 46,686 (34.7%) | $17,321 |
| 2020 | $0 | $0 | $0 | $6,077 | $3,284 | $1,225,442 | $855,973,763 | 140,859 | 46,793 (33.2%) | $18,293 |
| 2021 | $0 | $0 | $0 | $6,665 | $3,770 | $989,262 | $916,936,253 | 137,584 | 48,421 (35.2%) | $18,937 |
| 2022 | $0 | $0 | $0 | $7,334 | $4,412 | $1,661,065 | $1,009,413,151 | 137,627 | 51,228 (37.2%) | $19,704 |

**Table S4.**  Performance of all ensemble models on test dataset.

| **Ensemble model performance on test dataset** | | | | | | | | |
| --- | --- | --- | --- | --- | --- | --- | --- | --- |
| Predicting inpatient bed days category | | | | | | | | |
| Model characteristics | Accuracy  (95% CI) | Multi-class AUC (95% CI) | Correct predictions | Underpredictions | Overpredictions | Unnecessary predictions | Identified utilizers | Missed cases |
| Boosted trees with MARS as base learner | 0.6522  (0.6465 - 0.6579) | 0.6877  (0.6927 - 0.7255) | 30.5% | 23.2% | 24.1% | 31.3% | 77.8% | 22.2% |
| Boosted trees with MLP as base learner | 0.6685  (0.6629 - 0.6741) | 0.6832  (0.6875 - 0.7202) | 30.2% | 20.3% | 25.5% | 29.6% | 76.0% | 24.0% |
| Boosted trees with Logistic regression as base learner | 0.6495  (0.6438 - 0.6552) | 0.6696  (0.6896 - 0.722) | 30.2% | 20.0% | 26.0% | 31.7% | 76.1% | 23.9% |
| Boosted trees with Boosted trees as base learner | 0.6654  (0.6598 - 0.671) | 0.6776  (0.6936 - 0.7259) | 29.7% | 21.2% | 24.1% | 29.9% | 75.1% | 24.9% |
| SVM linear with MLP as base learner | 0.7300  (0.7246 - 0.7352) | 0.6758  (0.6757 - 0.7089) | 26.8% | 19.7% | 22.6% | 22.6% | 69.1% | 30.9% |
| SVM linear with MARS as base learner | 0.7455  (0.7403 - 0.7507) | 0.6828  (0.6738 - 0.7069) | 26.2% | 14.3% | 27.3% | 20.6% | 67.8% | 32.2% |
| SVM linear with Boost tree as base learner | 0.7305 (0.7252 - 0.7358) | 0.675  (0.6659 - 0.6986) | 25.8% | 14.2% | 27.1% | 22.3% | 67.1% | 32.9% |
| SVM linear with Logistic regression as base learner | 0.7339  (0.7286 - 0.7391) | 0.6656  (0.6698 - 0.7032) | 23.9% | 9.3% | 32.8% | 21.9% | 66.0% | 34.0% |
| Random forest with Logistic regression as base learner | 0.9010  (0.8974 - 0.9045) | 0.5357  (0.517 - 0.5331) | 3.8% | 2.4% | 3.3% | 1.6% | 9.4% | 90.6% |
| Random forest with Boost tree as base learner | 0.8907  (0.8869 - 0.8944) | 0.5319  (0.5076 - 0.5222) | 3.7% | 2.5% | 3.0% | 2.6% | 9.2% | 90.8% |
| Random forest with MLP as base learner | 0.9031  (0.8995 - 0.9065) | 0.5374  (0.5112 - 0.5254) | 3.3% | 2.9% | 2.7% | 1.3% | 9.0% | 91.0% |
| Random forest with MARS as base learner | 0.8949  (0.8912 - 0.8985) | 0.5373  (0.5045 - 0.517) | 3.3% | 2.8% | 3.0% | 1.8% | 9.1% | 90.9% |
|  | | | | | | | | |
| Predicting emergency department visits category | | | | | | | | |
| Model characteristics | Accuracy  (95% CI) | Multi-class AUC (95% CI) | Correct predictions | Underpredictions | Overpredictions | Unnecessary predictions | Identified utilizers | Missed cases |
| Boosted trees with MARS as base learner | 0.7457  (0.7405 - 0.7508) | 0.7601  (0.7301 - 0.7654) | 39.5% | 10.7% | 24.8% | 24.0% | 75.0% | 25.0% |
| Boosted trees with Logistic regression as base learner | 0.7205  (0.7151 - 0.7258) | 0.7569  (0.7294 - 0.7641) | 39.3% | 11.0% | 24.9% | 26.6% | 75.2% | 24.8% |
| Boosted trees with MLP as base learner | 0.7432  (0.7380 - 0.7484) | 0.7557  (0.731 - 0.7664) | 37.9% | 10.4% | 25.8% | 24.2% | 74.0% | 26.0% |
| Boosted trees with Boosted trees as base learner | 0.7425  (0.7372 - 0.7476) | 0.7527  (0.7016 - 0.7378) | 37.1% | 14.7% | 18.3% | 24.2% | 70.1% | 29.9% |
| SVM linear with MARS as base learner | 0.7849  (0.7800 - 0.7898) | 0.7722  (0.7316 - 0.7681) | 30.3% | 6.6% | 34.8% | 19.6% | 71.8% | 28.2% |
| SVM linear with MLP as base learner | 0.7883  (0.7834 - 0.7931) | 0.7959  (0.7164 - 0.7530) | 29.6% | 6.5% | 32.3% | 19.2% | 68.3% | 31.7% |
| SVM linear with Logistic regression as base learner | 0.7913  (0.7864 - 0.7961) | 0.7569  (0.7250 - 0.761) | 27.0% | 6.1% | 35.0% | 18.7% | 68.2% | 31.8% |
| SVM linear with Boost Tree as base learner | 0.7994  (0.7946 - 0.8042) | 0.7686  (0.6923 - 0.7295) | 27.0% | 6.9% | 29.5% | 17.8% | 63.3% | 36.7% |
| Random Forest with MLP as base learner | 0.9553  (0.9527 - 0.9577) | 0.6248  (0.5110 - 0.5258) | 4.4% | 1.7% | 1.5% | 0.8% | 7.6% | 92.4% |
| Random Forest with MARS as base learner | 0.9565  (0.9540 - 0.9589) | 0.5906  (0.5101 - 0.5246) | 4.2% | 1.9% | 1.4% | 0.7% | 7.6% | 92.4% |
| Random Forest with Logistic regression as base learner | 0.9559  (0.9534 - 0.9583) | 0.5973  (0.5136 - 0.5287) | 4.0% | 2.2% | 2.0% | 0.6% | 8.2% | 91.8% |
| Random Forest with Boost Tree as base learner | 0.9479  (0.9452 - 0.9505) | 0.5945  (0.5035 - 0.5162) | 2.4% | 2.0% | 1.5% | 1.3% | 5.8% | 94.2% |
| MARS: Multivariate Adaptive Regression Splines; MLP: Multi-Layered Perceptron; SVM: Support Vector Machines; AUC: Area under the receiver operator curve. | | | | | | | | |

**Table S5**. Detailed performance metrics for all models predicting inpatient bed days (test dataset).

| **Detailed model performance metrics** | | | | | | | | | | | | | |
| --- | --- | --- | --- | --- | --- | --- | --- | --- | --- | --- | --- | --- | --- |
| **Predicting inpatient bed days category** | | | | | | | | | | | | | |
| **Model: Random forest with Logistic Regression as the base learner** | | | | | | | | | | | | | |
| **Confusion Matrix (test dataset)** | | | | |  | **Model performance metrics (95% CI)** | | | | |  | **Additional metrics** | |
|  | Reference | | | |  | Multiclass AUC | 0.5357 (0.5170 - 0.5331) | | | |  | Correct predictions | 3.8% |
| Prediction | <7 days | 7 - 13 days | 14 - 29 days | ≥ 30 days |  | Accuracy | 0.9010 (0.8974 - 0.9045) | | | |  | Underpredictions | 2.4% |
| <7 days | 24436 | 873 | 746 | 537 |  |  |  |  |  |  |  | Overpredictions | 3.3% |
| 7 - 13 days | 121 | 11 | 16 | 12 |  | Class: | <7 days | 7 - 13 days | 14 - 29 days | ≥ 30 days |  | Unnecessary predictions | 1.6% |
| 14 - 29 days | 139 | 26 | 22 | 28 |  | Sensitivity | 0.9837 | 0.0118 | 0.0271 | 0.0899 |  | Identified utilizers | 9.4% |
| ≥ 30 days | 146 | 25 | 27 | 57 |  | Specificity | 0.0941 | 0.9943 | 0.9927 | 0.9926 |  | Missed cases | 90.6% |
|  |  |  |  |  |  | PPV | 0.9189 | 0.0688 | 0.1023 | 0.2235 |  |  |  |
|  |  |  |  |  |  | NPV | 0.3556 | 0.9659 | 0.9708 | 0.9786 |  |  |  |
|  |  |  |  |  |  | Prevalence | 0.9126 | 0.0343 | 0.0298 | 0.0233 |  |  |  |
|  |  |  |  |  |  | Detection Rate | 0.8977 | 0.0004 | 0.0008 | 0.0021 |  |  |  |
|  |  |  |  |  |  | Detection Prevalence | 0.9769 | 0.0059 | 0.0079 | 0.0094 |  |  |  |
|  |  |  |  |  |  | Balanced Accuracy | 0.5389 | 0.5030 | 0.5099 | 0.5412 |  |  |  |
|  |  |  |  |  |  |  |  |  |  |  |  |  |  |
| **Model: Boost tree with Logistic Regression as the base learner** | | | | | | | | | | | | | |
| **Confusion Matrix (test dataset)** | | | | |  | **Model performance metrics (95% CI)** | | | | |  | **Additional metrics** | |
|  | Reference | | | |  | Multiclass AUC | 0.6696 (0.6896 - 0.7220) | | | |  | Correct predictions | 30.2% |
| Prediction | <7 days | 7 - 13 days | 14 - 29 days | ≥ 30 days |  | Accuracy | 0.6495 (0.6438 - 0.6552) | | | |  | Underpredictions | 20.0% |
| <7 days | 16964 | 279 | 183 | 106 |  |  |  |  |  |  |  | Overpredictions | 26.0% |
| 7 - 13 days | 3457 | 227 | 182 | 121 |  | Class: | <7 days | 7 - 13 days | 14 - 29 days | ≥ 30 days |  | Unnecessary predictions | 31.7% |
| 14 - 29 days | 3053 | 262 | 257 | 173 |  | Sensitivity | 0.9676 | 0.0569 | 0.0686 | 0.1195 |  | Identified utilizers | 76.1% |
| ≥ 30 days | 1368 | 167 | 189 | 234 |  | Specificity | 0.1870 | 0.9695 | 0.9764 | 0.9842 |  | Missed cases | 23.9% |
|  |  |  |  |  |  | PPV | 0.6829 | 0.2428 | 0.3169 | 0.3691 |  |  |  |
|  |  |  |  |  |  | NPV | 0.7613 | 0.8570 | 0.8679 | 0.9352 |  |  |  |
|  |  |  |  |  |  | Prevalence | 0.6440 | 0.1465 | 0.1376 | 0.0719 |  |  |  |
|  |  |  |  |  |  | Detection Rate | 0.6232 | 0.0083 | 0.0094 | 0.0086 |  |  |  |
|  |  |  |  |  |  | Detection Prevalence | 0.9126 | 0.0343 | 0.0298 | 0.0233 |  |  |  |
|  |  |  |  |  |  | Balanced Accuracy | 0.5773 | 0.5132 | 0.5225 | 0.5518 |  |  |  |
|  |  |  |  |  |  |  |  |  |  |  |  |  |  |
| **Model: Linear SVM with Logistic Regression as the base learner** | | | | | | | | | | | | | |
| **Confusion Matrix (test dataset)** | | | | |  | **Model performance metrics (95% CI)** | | | | |  | **Additional metrics** | |
|  | Reference | | | |  | Multiclass AUC | 0.6656 (0.6698 - 0.7032) | | | |  | Correct predictions | 23.9% |
| Prediction | <7 days | 7 - 13 days | 14 - 29 days | ≥ 30 days |  | Accuracy | 0.7339 (0.7286 - 0.7391) | | | |  | Underpredictions | 9.3% |
| <7 days | 19410 | 397 | 263 | 150 |  |  |  |  |  |  |  | Overpredictions | 32.8% |
| 7 - 13 days | 1723 | 108 | 88 | 50 |  | Class: | <7 days | 7 - 13 days | 14 - 29 days | ≥ 30 days |  | Unnecessary predictions | 21.9% |
| 14 - 29 days | 1209 | 130 | 110 | 84 |  | Sensitivity | 0.7813 | 0.1155 | 0.1356 | 0.5521 |  | Identified utilizers | 66.0% |
| ≥ 30 days | 2500 | 300 | 350 | 350 |  | Specificity | 0.6597 | 0.9292 | 0.9461 | 0.8815 |  | Missed cases | 34.0% |
|  |  |  |  |  |  | PPV | 0.9599 | 0.0549 | 0.0718 | 0.1000 |  |  |  |
|  |  |  |  |  |  | NPV | 0.2242 | 0.9673 | 0.9727 | 0.9880 |  |  |  |
|  |  |  |  |  |  | Prevalence | 0.9126 | 0.0343 | 0.0298 | 0.0233 |  |  |  |
|  |  |  |  |  |  | Detection | 0.7130 | 0.0040 | 0.0040 | 0.0129 |  |  |  |
|  |  |  |  |  |  | Detection | 0.7428 | 0.0723 | 0.0563 | 0.1286 |  |  |  |
|  |  |  |  |  |  | Balanced | 0.7205 | 0.5224 | 0.5409 | 0.7168 |  |  |  |
|  |  |  |  |  |  |  |  |  |  |  |  |  |  |
| **Model: Random forest with boosted tree as the base learner** | | | | | | | | | | | | | |
| **Confusion Matrix (test dataset)** | | | | |  | **Model performance metrics (95% CI)** | | | | |  | **Additional metrics** | |
|  | Reference | | | |  | Multiclass AUC | 0.5319 (0.5076 - 0.5222) | | | |  | Correct predictions | 3.7% |
| Prediction | <7 days | 7 - 13 days | 14 - 29 days | ≥ 30 days |  | Accuracy | 0.8907 (0.8869 - 0.8944) | | | |  | Underpredictions | 2.5% |
| <7 days | 24157 | 914 | 694 | 585 |  |  |  |  |  |  |  | Overpredictions | 3.0% |
| 7 - 13 days | 245 | 17 | 12 | 12 |  | Class: | <7 days | 7 - 13 days | 14 - 29 days | ≥ 30 days |  | Unnecessary predictions | 2.6% |
| 14 - 29 days | 221 | 14 | 27 | 37 |  | Sensitivity | 0.9738 | 0.0176 | 0.0351 | 0.0663 |  | Identified utilizers | 9.2% |
| ≥ 30 days | 183 | 23 | 36 | 45 |  | Specificity | 0.0923 | 0.9898 | 0.9897 | 0.9909 |  | Missed cases | 90.8% |
|  |  |  |  |  |  | PPV | 0.9168 | 0.0594 | 0.0903 | 0.1568 |  |  |  |
|  |  |  |  |  |  | NPV | 0.2557 | 0.9647 | 0.9724 | 0.9765 |  |  |  |
|  |  |  |  |  |  | Prevalence | 0.9112 | 0.0356 | 0.0282 | 0.0249 |  |  |  |
|  |  |  |  |  |  | Detection Rate | 0.8874 | 0.0006 | 0.0010 | 0.0017 |  |  |  |
|  |  |  |  |  |  | Detection Prevalence | 0.9680 | 0.0105 | 0.0110 | 0.0105 |  |  |  |
|  |  |  |  |  |  | Balanced Accuracy | 0.5331 | 0.5037 | 0.5124 | 0.5286 |  |  |  |
|  |  |  |  |  |  |  |  |  |  |  |  |  |  |
| **Model: Boosted tree with boosted tree as the base learner** | | | | | | | | | | | | | |
| **Confusion Matrix (test dataset)** | | | | |  | **Model performance metrics (95% CI)** | | | | |  | **Additional metrics** | |
|  | Reference | | | |  | Multiclass AUC | 0.6776 (0.6936 - 0.7259) | | | |  | Correct predictions | 29.7% |
| Prediction | <7 days | 7 - 13 days | 14 - 29 days | ≥ 30 days |  | Accuracy | 0.6654 (0.6598 - 0.6710) | | | |  | Underpredictions | 21.2% |
| <7 days | 17396 | 305 | 183 | 114 |  |  |  |  |  |  |  | Overpredictions | 24.1% |
| 7 - 13 days | 4019 | 265 | 189 | 133 |  | Class: | <7 days | 7 - 13 days | 14 - 29 days | ≥ 30 days |  | Unnecessary predictions | 29.9% |
| 14 - 29 days | 2229 | 232 | 212 | 191 |  | Sensitivity | 0.7013 | 0.2738 | 0.2757 | 0.3549 |  | Identified utilizers | 75.1% |
| ≥ 30 days | 1162 | 166 | 185 | 241 |  | Specificity | 0.7508 | 0.8347 | 0.8997 | 0.9430 |  | Missed cases | 24.9% |
|  |  |  |  |  |  | PPV | 0.9666 | 0.0575 | 0.0740 | 0.1374 |  |  |  |
|  |  |  |  |  |  | NPV | 0.1967 | 0.9689 | 0.9771 | 0.9828 |  |  |  |
|  |  |  |  |  |  | Prevalence | 0.9112 | 0.0356 | 0.0282 | 0.0249 |  |  |  |
|  |  |  |  |  |  | Detection Rate | 0.6390 | 0.0097 | 0.0078 | 0.0089 |  |  |  |
|  |  |  |  |  |  | Detection Prevalence | 0.6612 | 0.1692 | 0.1052 | 0.0644 |  |  |  |
|  |  |  |  |  |  | Balanced Accuracy | 0.7261 | 0.5542 | 0.5877 | 0.6490 |  |  |  |
|  |  |  |  |  |  |  |  |  |  |  |  |  |  |
| **Model: Linear SVM with boosted tree as the base learner** | | | | | | | | | | | | | |
| **Confusion Matrix (test dataset)** | | | | |  | **Model performance metrics (95% CI)** | | | | |  | **Additional metrics** | |
|  | Reference | | | |  | Multiclass AUC | 0.6750 (0.6659 - 0.6986) | | | |  | Correct predictions | 25.8% |
| Prediction | <7 days | 7 - 13 days | 14 - 29 days | ≥ 30 days |  | Accuracy | 0.7305 (0.7252 - 0.7358) | | | |  | Underpredictions | 14.2% |
| <7 days | 19263 | 411 | 238 | 145 |  |  |  |  |  |  |  | Overpredictions | 27.1% |
| 7 - 13 days | 1525 | 102 | 69 | 66 |  | Class: | <7 days | 7 - 13 days | 14 - 29 days | ≥ 30 days |  | Unnecessary predictions | 22.3% |
| 14 - 29 days | 2703 | 302 | 262 | 209 |  | Sensitivity | 0.7765 | 0.1054 | 0.3407 | 0.3814 |  | Identified utilizers | 67.1% |
| ≥ 30 days | 1315 | 153 | 200 | 259 |  | Specificity | 0.6714 | 0.9368 | 0.8785 | 0.9372 |  | Missed cases | 32.9% |
|  |  |  |  |  |  | PPV | 0.9604 | 0.0579 | 0.0754 | 0.1344 |  |  |  |
|  |  |  |  |  |  | NPV | 0.2264 | 0.9660 | 0.9786 | 0.9834 |  |  |  |
|  |  |  |  |  |  | Prevalence | 0.9112 | 0.0356 | 0.0282 | 0.0249 |  |  |  |
|  |  |  |  |  |  | Detection Rate | 0.7076 | 0.0037 | 0.0096 | 0.0095 |  |  |  |
|  |  |  |  |  |  | Detection Prevalence | 0.7368 | 0.0647 | 0.1277 | 0.0708 |  |  |  |
|  |  |  |  |  |  | Balanced Accuracy | 0.7240 | 0.5211 | 0.6096 | 0.6593 |  |  |  |
|  |  |  |  |  |  |  |  |  |  |  |  |  |  |
| **Model: Random forest with MARS as the base learner** | | | | | | | | | | | | | |
| **Confusion Matrix (test dataset)** | | | | |  | **Model performance metrics (95% CI)** | | | | |  | **Additional metrics** | |
|  | Reference | | | |  | Multiclass AUC | 0.5373 (0.5045 - 0.5170) | | | |  | Correct predictions | 3.3% |
| Prediction | <7 days | 7 - 13 days | 14 - 29 days | ≥ 30 days |  | Accuracy | 0.8949 (0.8912 - 0.8985) | | | |  | Underpredictions | 2.8% |
| <7 days | 24279 | 909 | 766 | 602 |  |  |  |  |  |  |  | Overpredictions | 3.0% |
| 7 - 13 days | 126 | 7 | 14 | 24 |  | Class: | <7 days | 7 - 13 days | 14 - 29 days | ≥ 30 days |  | Unnecessary predictions | 1.8% |
| 14 - 29 days | 147 | 11 | 27 | 31 |  | Sensitivity | 0.9822 | 0.0074 | 0.0317 | 0.0681 |  | Identified utilizers | 9.1% |
| ≥ 30 days | 166 | 19 | 46 | 48 |  | Specificity | 0.0907 | 0.9938 | 0.9928 | 0.9913 |  | Missed cases | 90.9% |
|  |  |  |  |  |  | PPV | 0.9143 | 0.0409 | 0.1250 | 0.1720 |  |  |  |
|  |  |  |  |  |  | NPV | 0.3408 | 0.9653 | 0.9694 | 0.9756 |  |  |  |
|  |  |  |  |  |  | Prevalence | 0.9080 | 0.0348 | 0.0313 | 0.0259 |  |  |  |
|  |  |  |  |  |  | Detection Rate | 0.8919 | 0.0003 | 0.0010 | 0.0018 |  |  |  |
|  |  |  |  |  |  | Detection Prevalence | 0.9755 | 0.0063 | 0.0079 | 0.0102 |  |  |  |
|  |  |  |  |  |  | Balanced Accuracy | 0.5365 | 0.5006 | 0.5122 | 0.5297 |  |  |  |
|  |  |  |  |  |  |  |  |  |  |  |  |  |  |
| **Model: Boost tree forest with MARS as the base learner** | | | | | | | | | | | | | |
| **Confusion Matrix (test dataset)** | | | | |  | **Model performance metrics (95% CI)** | | | | |  | **Additional metrics** | |
|  | Reference | | | |  | Multiclass AUC | 0.6877 (0.6927 - 0.7255) | | | |  | Correct predictions | 30.5% |
| Prediction | <7 days | 7 - 13 days | 14 - 29 days | ≥ 30 days |  | Accuracy | 0.6522 (0.6465 - 0.6579) | | | |  | Underpredictions | 23.2% |
| <7 days | 16991 | 291 | 168 | 97 |  |  |  |  |  |  |  | Overpredictions | 24.1% |
| 7 - 13 days | 4280 | 252 | 239 | 141 |  | Class: | <7 days | 7 - 13 days | 14 - 29 days | ≥ 30 days |  | Unnecessary predictions | 31.3% |
| 14 - 29 days | 2166 | 241 | 245 | 201 |  | Sensitivity | 0.6874 | 0.2664 | 0.2872 | 0.3773 |  | Identified utilizers | 77.8% |
| ≥ 30 days | 1281 | 162 | 201 | 266 |  | Specificity | 0.7780 | 0.8227 | 0.9011 | 0.9380 |  | Missed cases | 22.2% |
|  |  |  |  |  |  | PPV | 0.9683 | 0.0513 | 0.0859 | 0.1393 |  |  |  |
|  |  |  |  |  |  | NPV | 0.2013 | 0.9689 | 0.9751 | 0.9827 |  |  |  |
|  |  |  |  |  |  | Prevalence | 0.9080 | 0.0348 | 0.0313 | 0.0259 |  |  |  |
|  |  |  |  |  |  | Detection Rate | 0.6242 | 0.0093 | 0.0090 | 0.0098 |  |  |  |
|  |  |  |  |  |  | Detection Prevalence | 0.6446 | 0.1804 | 0.1048 | 0.0702 |  |  |  |
|  |  |  |  |  |  | Balanced Accuracy | 0.7327 | 0.5445 | 0.5942 | 0.6577 |  |  |  |
|  |  |  |  |  |  |  |  |  |  |  |  |  |  |
| **Model: Linear SVM with MARS as the base learner** | | | | | | | | | | | | | |
| **Confusion Matrix (test dataset)** | | | | |  | **Model performance metrics (95% CI)** | | | | |  | **Additional metrics** | |
|  | Reference | | | |  | Multiclass AUC | 0.6828 (0.6738 - 0.7069) | | | |  | Correct predictions | 26.2% |
| Prediction | <7 days | 7 - 13 days | 14 - 29 days | ≥ 30 days |  | Accuracy | 0.7455 (0.7403 - 0.7507) | | | |  | Underpredictions | 14.3% |
| <7 days | 19638 | 405 | 262 | 140 |  |  |  |  |  |  |  | Overpredictions | 27.3% |
| 7 - 13 days | 2032 | 158 | 140 | 98 |  | Class: | <7 days | 7 - 13 days | 14 - 29 days | ≥ 30 days |  | Unnecessary predictions | 20.6% |
| 14 - 29 days | 1305 | 151 | 150 | 119 |  | Sensitivity | 0.7945 | 0.1670 | 0.1759 | 0.4936 |  | Identified utilizers | 67.8% |
| ≥ 30 days | 1743 | 232 | 301 | 348 |  | Specificity | 0.6777 | 0.9136 | 0.9403 | 0.9142 |  | Missed cases | 32.2% |
|  |  |  |  |  |  | PPV | 0.9605 | 0.0651 | 0.0870 | 0.1326 |  |  |  |
|  |  |  |  |  |  | NPV | 0.2504 | 0.9682 | 0.9724 | 0.9855 |  |  |  |
|  |  |  |  |  |  | Prevalence | 0.9080 | 0.0348 | 0.0313 | 0.0259 |  |  |  |
|  |  |  |  |  |  | Detection Rate | 0.7214 | 0.0058 | 0.0055 | 0.0128 |  |  |  |
|  |  |  |  |  |  | Detection Prevalence | 0.7510 | 0.0892 | 0.0634 | 0.0964 |  |  |  |
|  |  |  |  |  |  | Balanced Accuracy | 0.7361 | 0.5403 | 0.5581 | 0.7039 |  |  |  |
|  |  |  |  |  |  |  |  |  |  |  |  |  |  |
|  |  |  |  |  |  |  |  |  |  |  |  |  |  |
| **Model: Random forest with MLP as the base learner** | | | | | | | | | | | | | |
| **Confusion Matrix (test dataset)** | | | | |  | **Model performance metrics (95% CI)** | | | | |  | **Additional metrics** | |
|  | Reference | | | |  | Multiclass AUC | 0.5374 (0.5112 - 0.5254) | | | |  | Correct predictions | 3.3% |
| Prediction | <7 days | 7 - 13 days | 14 - 29 days | ≥ 30 days |  | Accuracy | 0.9031 (0.8995 - 0.9065) | | | |  | Underpredictions | 2.9% |
| <7 days | 24503 | 877 | 734 | 566 |  |  |  |  |  |  |  | Overpredictions | 2.7% |
| 7 - 13 days | 75 | 14 | 15 | 24 |  | Class: | <7 days | 7 - 13 days | 14 - 29 days | ≥ 30 days |  | Unnecessary predictions | 1.3% |
| 14 - 29 days | 101 | 11 | 20 | 31 |  | Sensitivity | 0.9868 | 0.0152 | 0.0250 | 0.0690 |  | Identified utilizers | 9.0% |
| ≥ 30 days | 152 | 21 | 32 | 46 |  | Specificity | 0.0895 | 0.9957 | 0.9946 | 0.9923 |  | Missed cases | 91.0% |
|  |  |  |  |  |  | PPV | 0.9184 | 0.1094 | 0.1227 | 0.1833 |  |  |  |
|  |  |  |  |  |  | NPV | 0.3948 | 0.9665 | 0.9711 | 0.9770 |  |  |  |
|  |  |  |  |  |  | Prevalence | 0.9122 | 0.0339 | 0.0294 | 0.0245 |  |  |  |
|  |  |  |  |  |  | Detection Rate | 0.9001 | 0.0005 | 0.0007 | 0.0017 |  |  |  |
|  |  |  |  |  |  | Detection Prevalence | 0.9801 | 0.0047 | 0.0060 | 0.0092 |  |  |  |
|  |  |  |  |  |  | Balanced Accuracy | 0.5381 | 0.5054 | 0.5098 | 0.5306 |  |  |  |
|  |  |  |  |  |  |  |  |  |  |  |  |  |  |
| **Model: Boost tree with MLP as the base learner** | | | | | | | | | | | | | |
| **Confusion Matrix (test dataset)** | | | | |  | **Model performance metrics (95% CI)** | | | | |  | **Additional metrics** | |
|  | Reference | | | |  | Multiclass AUC | 0.6832 (0.6875 - 0.7202) | | | |  | Correct predictions | 30.2% |
| Prediction | <7 days | 7 - 13 days | 14 - 29 days | ≥ 30 days |  | Accuracy | 0.6685 (0.6629 - 0.6741) | | | |  | Underpredictions | 20.3% |
| <7 days | 17476 | 295 | 179 | 101 |  |  |  |  |  |  |  | Overpredictions | 25.5% |
| 7 - 13 days | 3158 | 209 | 167 | 110 |  | Class: | <7 days | 7 - 13 days | 14 - 29 days | ≥ 30 days |  | Unnecessary predictions | 29.6% |
| 14 - 29 days | 2925 | 270 | 265 | 208 |  | Sensitivity | 0.7038 | 0.2264 | 0.3308 | 0.3718 |  | Identified utilizers | 76.0% |
| ≥ 30 days | 1272 | 149 | 190 | 248 |  | Specificity | 0.7595 | 0.8694 | 0.8712 | 0.9393 |  | Missed cases | 24.0% |
|  |  |  |  |  |  | PPV | 0.9681 | 0.0574 | 0.0722 | 0.1334 |  |  |  |
|  |  |  |  |  |  | NPV | 0.1980 | 0.9697 | 0.9772 | 0.9835 |  |  |  |
|  |  |  |  |  |  | Prevalence | 0.9122 | 0.0339 | 0.0294 | 0.0245 |  |  |  |
|  |  |  |  |  |  | Detection Rate | 0.6420 | 0.0077 | 0.0097 | 0.0091 |  |  |  |
|  |  |  |  |  |  | Detection Prevalence | 0.6631 | 0.1339 | 0.1347 | 0.0683 |  |  |  |
|  |  |  |  |  |  | Balanced Accuracy | 0.7317 | 0.5479 | 0.6010 | 0.6556 |  |  |  |
|  |  |  |  |  |  |  |  |  |  |  |  |  |  |
| **Model: Linear SVM tree with MLP as the base learner** | | | | | | | | | | | | | |
| **Confusion Matrix (test dataset)** | | | | |  | **Model performance metrics (95% CI)** | | | | |  | **Additional metrics** | |
|  | Reference | | | |  | Multiclass AUC | 0.6758 (0.6757 - 0.7089) | | | |  | Correct predictions | 26.8% |
| Prediction | <7 days | 7 - 13 days | 14 - 29 days | ≥ 30 days |  | Accuracy | 0.7300 (0.7246 - 0.7352) | | | |  | Underpredictions | 19.7% |
| <7 days | 19231 | 372 | 234 | 134 |  |  |  |  |  |  |  | Overpredictions | 22.6% |
| 7 - 13 days | 1533 | 98 | 81 | 50 |  | Class: | <7 days | 7 - 13 days | 14 - 29 days | ≥ 30 days |  | Unnecessary predictions | 22.6% |
| 14 - 29 days | 3443 | 382 | 398 | 339 |  | Sensitivity | 0.7745 | 0.1062 | 0.4969 | 0.2159 |  | Identified utilizers | 69.1% |
| ≥ 30 days | 624 | 71 | 88 | 144 |  | Specificity | 0.6905 | 0.9367 | 0.8424 | 0.9705 |  | Missed cases | 30.9% |
|  |  |  |  |  |  | PPV | 0.9629 | 0.0556 | 0.0872 | 0.1553 |  |  |  |
|  |  |  |  |  |  | NPV | 0.2277 | 0.9676 | 0.9822 | 0.9801 |  |  |  |
|  |  |  |  |  |  | Prevalence | 0.9122 | 0.0339 | 0.0294 | 0.0245 |  |  |  |
|  |  |  |  |  |  | Detection Rate | 0.7065 | 0.0036 | 0.0146 | 0.0053 |  |  |  |
|  |  |  |  |  |  | Detection Prevalence | 0.7336 | 0.0647 | 0.1676 | 0.0341 |  |  |  |
|  |  |  |  |  |  | Balanced Accuracy | 0.7325 | 0.5215 | 0.6696 | 0.5932 |  |  |  |
| MARS: Multivariate Adaptive Regression Splines; MLP: Multi-Layered Perceptron; SVM: Support Vector Machines; AUC: Area under the receiver operator curve; PPV: Positive predictive value; NPV: Negative predictive value. | | | | | | | | | | | | | |

**Table S6**. Detailed model performance metrics for predicting emergency department visits (test dataset).

| **Detailed model performance metrics** | | | | | | | | | | | | | |
| --- | --- | --- | --- | --- | --- | --- | --- | --- | --- | --- | --- | --- | --- |
| **Predicting emergency department visits category** | | | | | | | | | | | | | |
| **Model: Random forest with Logistic Regression as the base learner** | | | | | | | | | | | | | |
| **Confusion Matrix (test dataset)** | | | | |  | **Model performance metrics (95% CI)** | | | | |  | **Additional metrics** | |
|  | Reference | | | |  | Multiclass AUC | 0.5973 (0.5136 - 0.5287) | | | |  | Correct | 4.0% |
| Prediction | < 3 visits | 3 - 4 visits | 5 - 9 visits | ≥ 10 visits |  | Accuracy | 0.9559 (0.9534 - 0.9583) | | | |  | Underprediction | 2.2% |
| < 3 visits | 25979 | 742 | 214 | 32 |  |  |  |  |  |  |  | Overprediction | 2.0% |
| 3 - 4 visits | 103 | 23 | 14 | 4 |  | Class: | < 3 visits | 3 - 4 visits | 5 - 9 visits | ≥ 10 visits |  | Unnecessary predictions | 0.6% |
| 5 - 9 visits | 53 | 11 | 12 | 6 |  | Sensitivity | 0.9936 | 0.0295 | 0.0488 | 0.1600 |  | Intervention | 8.2% |
| ≥ 10 visits | 11 | 4 | 6 | 8 |  | Specificity | 0.0818 | 0.9954 | 0.9974 | 0.9992 |  | Missed cases | 91.8% |
|  |  |  |  |  |  | PPV | 0.9634 | 0.1597 | 0.1463 | 0.2759 |  |  |  |
|  |  |  |  |  |  | NPV | 0.3451 | 0.9720 | 0.9914 | 0.9985 |  |  |  |
|  |  |  |  |  |  | Prevalence | 0.9605 | 0.0287 | 0.0090 | 0.0018 |  |  |  |
|  |  |  |  |  |  | Detection Rate | 0.9543 | 0.0008 | 0.0004 | 0.0003 |  |  |  |
|  |  |  |  |  |  | Detection Prevalence | 0.9906 | 0.0053 | 0.0030 | 0.0011 |  |  |  |
|  |  |  |  |  |  | Balanced Accuracy | 0.5377 | 0.5125 | 0.5231 | 0.5796 |  |  |  |
|  |  |  |  |  |  |  |  |  |  |  |  |  |  |
| **Model: Boosted tree with Logistic Regression as the base learner** | | | | | | | | | | | | | |
| **Confusion Matrix (test dataset)** | | | | |  | **Model performance metrics (95% CI)** | | | | |  | **Additional metrics** | |
|  | Reference | | | |  | Multiclass AUC | 0.7569 (0.7294 - 0.7641) | | | |  | Correct | 39.3% |
| Prediction | < 3 visits | 3 - 4 visits | 5 - 9 visits | ≥ 10 visits |  | Accuracy | 0.7205 (0.7151 - 0.7258) | | | |  | Underprediction | 11.0% |
| < 3 visits | 19191 | 220 | 43 | 4 |  |  |  |  |  |  |  | Overprediction | 24.9% |
| 3 - 4 visits | 5475 | 321 | 96 | 10 |  | Class: | < 3 visits | 3 - 4 visits | 5 - 9 visits | ≥ 10 visits |  | Unnecessary predictions | 26.6% |
| 5 - 9 visits | 1293 | 200 | 78 | 12 |  | Sensitivity | 0.7340 | 0.4115 | 0.3171 | 0.4800 |  | Intervention | 75.2% |
| ≥ 10 visits | 187 | 39 | 29 | 24 |  | Specificity | 0.7519 | 0.7889 | 0.9442 | 0.9906 |  | Missed cases | 24.8% |
|  |  |  |  |  |  | PPV | 0.9863 | 0.0544 | 0.0493 | 0.0860 |  |  |  |
|  |  |  |  |  |  | NPV | 0.1042 | 0.9785 | 0.9934 | 0.9990 |  |  |  |
|  |  |  |  |  |  | Prevalence | 0.9605 | 0.0287 | 0.0090 | 0.0018 |  |  |  |
|  |  |  |  |  |  | Detection | 0.7050 | 0.0118 | 0.0029 | 0.0009 |  |  |  |
|  |  |  |  |  |  | Detection | 0.7148 | 0.2168 | 0.0582 | 0.0102 |  |  |  |
|  |  |  |  |  |  | Balanced | 0.7429 | 0.6002 | 0.6306 | 0.7353 |  |  |  |
|  |  |  |  |  |  |  |  |  |  |  |  |  |  |
| **Model: Linear SVM with Logistic Regression as the base learner** | | | | | | | | | | | | | |
| **Confusion Matrix (test dataset)** | | | | |  | **Model performance metrics (95% CI)** | | | | |  | **Additional metrics** | |
|  | Reference | | | |  | Multiclass AUC | 0.7569 (0.7250 - 0.7610) | | | |  | Correct | 27.0% |
| Prediction | < 3 visits | 3 - 4 visits | 5 - 9 visits | ≥ 10 visits |  | Accuracy | 0.7913 (0.7864 - 0.7961) | | | |  | Underprediction | 6.1% |
| < 3 visits | 21250 | 279 | 59 | 4 |  |  |  |  |  |  |  | Overprediction | 35.0% |
| 3 - 4 visits | 3100 | 194 | 51 | 7 |  | Class: | < 3 visits | 3 - 4 visits | 5 - 9 visits | ≥ 10 visits |  | Unnecessary predictions | 18.7% |
| 5 - 9 visits | 1243 | 197 | 66 | 8 |  | Sensitivity | 0.8127 | 0.2487 | 0.2683 | 0.6200 |  | Intervention | 68.2% |
| ≥ 10 visits | 553 | 110 | 70 | 31 |  | Specificity | 0.6822 | 0.8806 | 0.9463 | 0.9730 |  | Missed cases | 31.8% |
|  |  |  |  |  |  | PPV | 0.9842 | 0.0579 | 0.0436 | 0.0406 |  |  |  |
|  |  |  |  |  |  | NPV | 0.1304 | 0.9755 | 0.9930 | 0.9993 |  |  |  |
|  |  |  |  |  |  | Prevalence | 0.9605 | 0.0287 | 0.0090 | 0.0018 |  |  |  |
|  |  |  |  |  |  | Detection | 0.7806 | 0.0071 | 0.0024 | 0.0011 |  |  |  |
|  |  |  |  |  |  | Detection | 0.7932 | 0.1231 | 0.0556 | 0.0281 |  |  |  |
|  |  |  |  |  |  | Balanced | 0.7474 | 0.5646 | 0.6073 | 0.7965 |  |  |  |
|  |  |  |  |  |  |  |  |  |  |  |  |  |  |
| **Model: Random forest with Boosted Tree as the base learner** | | | | | | | | | | | | | |
| **Confusion Matrix (test dataset)** | | | | |  | **Model performance metrics (95% CI)** | | | | |  | **Additional metrics** | |
|  | Reference | | | |  | Multiclass AUC | 0.5945 (0.5035 - 0.5162) | | | |  | Correct | 2.4% |
| Prediction | < 3 visits | 3 - 4 visits | 5 - 9 visits | ≥ 10 visits |  | Accuracy | 0.9479 (0.9452 - 0.9505) | | | |  | Underprediction | 2.0% |
| < 3 visits | 25778 | 740 | 269 | 29 |  |  |  |  |  |  |  | Overprediction | 1.5% |
| 3 - 4 visits | 254 | 11 | 12 | 3 |  | Class: | < 3 visits | 3 - 4 visits | 5 - 9 visits | ≥ 10 visits |  | Unnecessary predictions | 1.3% |
| 5 - 9 visits | 76 | 12 | 8 | 7 |  | Sensitivity | 0.9869 | 0.0144 | 0.0275 | 0.1522 |  | Intervention | 5.8% |
| ≥ 10 visits | 12 | 2 | 2 | 7 |  | Specificity | 0.0581 | 0.9898 | 0.9965 | 0.9994 |  | Missed cases | 94.2% |
|  |  |  |  |  |  | PPV | 0.9613 | 0.0393 | 0.0777 | 0.3043 |  |  |  |
|  |  |  |  |  |  | NPV | 0.1576 | 0.9720 | 0.9896 | 0.9986 |  |  |  |
|  |  |  |  |  |  | Prevalence | 0.9595 | 0.0281 | 0.0107 | 0.0017 |  |  |  |
|  |  |  |  |  |  | Detection Rate | 0.9470 | 0.0004 | 0.0003 | 0.0003 |  |  |  |
|  |  |  |  |  |  | Detection Prevalence | 0.9851 | 0.0103 | 0.0038 | 0.0008 |  |  |  |
|  |  |  |  |  |  | Balanced Accuracy | 0.5225 | 0.5021 | 0.5120 | 0.5758 |  |  |  |
|  |  |  |  |  |  |  |  |  |  |  |  |  |  |
| **Model: Boosted Tree with Boosted Tree as the base learner** | | | | | | | | | | | | | |
| **Confusion Matrix (test dataset)** | | | | |  | **Model performance metrics (95% CI)** | | | | |  | **Additional metrics** | |
|  | Reference | | | |  | Multiclass AUC | 0.7527 (0.7016 - 0.7378) | | | |  | Correct | 37.1% |
| Prediction | < 3 visits | 3 - 4 visits | 5 - 9 visits | ≥ 10 visits |  | Accuracy | 0.7425 (0.7372 - 0.7476) | | | |  | Underprediction | 14.7% |
| < 3 visits | 19802 | 266 | 61 | 2 |  |  |  |  |  |  |  | Overprediction | 18.3% |
| 3 - 4 visits | 5129 | 313 | 130 | 13 |  | Class: | < 3 visits | 3 - 4 visits | 5 - 9 visits | ≥ 10 visits |  | Unnecessary predictions | 24.2% |
| 5 - 9 visits | 1054 | 166 | 84 | 19 |  | Sensitivity | 0.7581 | 0.4092 | 0.2887 | 0.2609 |  | Intervention | 70.1% |
| ≥ 10 visits | 135 | 20 | 16 | 12 |  | Specificity | 0.7015 | 0.8007 | 0.9540 | 0.9937 |  | Missed cases | 29.9% |
|  |  |  |  |  |  | PPV | 0.9837 | 0.0560 | 0.0635 | 0.0656 |  |  |  |
|  |  |  |  |  |  | NPV | 0.1090 | 0.9791 | 0.9920 | 0.9987 |  |  |  |
|  |  |  |  |  |  | Prevalence | 0.9595 | 0.0281 | 0.0107 | 0.0017 |  |  |  |
|  |  |  |  |  |  | Detection | 0.7274 | 0.0115 | 0.0031 | 0.0004 |  |  |  |
|  |  |  |  |  |  | Detection | 0.7395 | 0.2052 | 0.0486 | 0.0067 |  |  |  |
|  |  |  |  |  |  | Balanced | 0.7298 | 0.6049 | 0.6213 | 0.6273 |  |  |  |
|  |  |  |  |  |  |  |  |  |  |  |  |  |  |
| **Model: Linear SVM with Boosted Tree as the base learner** | | | | | | | | | | | | | |
| **Confusion Matrix (test dataset)** | | | | |  | **Model performance metrics (95% CI)** | | | | |  | **Additional metrics** | |
|  | Reference | | | |  | Multiclass AUC | 0.7686 (0.6923 - 0.7295) | | | |  | Correct | 27.0% |
| Prediction | < 3 visits | 3 - 4 visits | 5 - 9 visits | ≥ 10 visits |  | Accuracy | 0.7994 (0.7946 - 0.8042) | | | |  | Underprediction | 6.9% |
| < 3 visits | 21465 | 325 | 77 | 2 |  |  |  |  |  |  |  | Overprediction | 29.5% |
| 3 - 4 visits | 2769 | 170 | 56 | 5 |  | Class: | < 3 visits | 3 - 4 visits | 5 - 9 visits | ≥ 10 visits |  | Unnecessary predictions | 17.8% |
| 5 - 9 visits | 1602 | 193 | 103 | 15 |  | Sensitivity | 0.8218 | 0.2222 | 0.3540 | 0.5217 |  | Intervention | 63.3% |
| ≥ 10 visits | 284 | 77 | 55 | 24 |  | Specificity | 0.6334 | 0.8930 | 0.9328 | 0.9847 |  | Missed cases | 36.7% |
|  |  |  |  |  |  | PPV | 0.9815 | 0.0567 | 0.0538 | 0.0545 |  |  |  |
|  |  |  |  |  |  | NPV | 0.1304 | 0.9754 | 0.9926 | 0.9992 |  |  |  |
|  |  |  |  |  |  | Prevalence | 0.9595 | 0.0281 | 0.0107 | 0.0017 |  |  |  |
|  |  |  |  |  |  | Detection | 0.7885 | 0.0062 | 0.0038 | 0.0009 |  |  |  |
|  |  |  |  |  |  | Detection | 0.8034 | 0.1102 | 0.0703 | 0.0162 |  |  |  |
|  |  |  |  |  |  | Balanced | 0.7276 | 0.5576 | 0.6434 | 0.7532 |  |  |  |
|  |  |  |  |  |  |  |  |  |  |  |  |  |  |
| **Model: Random Forest with MARS as the base learner** | | | | | | | | | | | | | |
| **Confusion Matrix (test dataset)** | | | | |  | **Model performance metrics (95% CI)** | | | | |  | **Additional metrics** | |
|  | Reference | | | |  | Multiclass AUC | 0.5906 (0.5101 - 0.5246) | | | |  | Correct | 4.2% |
| Prediction | < 3 visits | 3 - 4 visits | 5 - 9 visits | ≥ 10 visits |  | Accuracy | 0.9565 (0.9540 - 0.9589) | | | |  | Underprediction | 1.9% |
| < 3 visits | 25993 | 708 | 223 | 29 |  |  |  |  |  |  |  | Overprediction | 1.4% |
| 3 - 4 visits | 128 | 18 | 16 | 1 |  | Class: | < 3 visits | 3 - 4 visits | 5 - 9 visits | ≥ 10 visits |  | Unnecessary predictions | 0.7% |
| 5 - 9 visits | 49 | 8 | 16 | 3 |  | Sensitivity | 0.9927 | 0.0244 | 0.0623 | 0.2326 |  | Intervention | 7.6% |
| ≥ 10 visits | 13 | 5 | 2 | 10 |  | Specificity | 0.0760 | 0.9945 | 0.9978 | 0.9993 |  | Missed cases | 92.4% |
|  |  |  |  |  |  | PPV | 0.9644 | 0.1104 | 0.2105 | 0.3333 |  |  |  |
|  |  |  |  |  |  | NPV | 0.2937 | 0.9734 | 0.9911 | 0.9988 |  |  |  |
|  |  |  |  |  |  | Prevalence | 0.9618 | 0.0271 | 0.0094 | 0.0016 |  |  |  |
|  |  |  |  |  |  | Detection | 0.9549 | 0.0007 | 0.0006 | 0.0004 |  |  |  |
|  |  |  |  |  |  | Detection | 0.9901 | 0.0060 | 0.0028 | 0.0011 |  |  |  |
|  |  |  |  |  |  | Balanced | 0.5344 | 0.5094 | 0.5300 | 0.6159 |  |  |  |
|  |  |  |  |  |  |  |  |  |  |  |  |  |  |
| **Model: Boosted Tree with MARS as the base learner** | | | | | | | | | | | | | |
| **Confusion Matrix (test dataset)** | | | | |  | **Model performance metrics (95% CI)** | | | | |  | **Additional metrics** | |
|  | Reference | | | |  | Multiclass AUC | 0.7601 (0.7301 - 0.7654) | | | |  | Correct | 39.5% |
| Prediction | < 3 visits | 3 - 4 visits | 5 - 9 visits | ≥ 10 visits |  | Accuracy | 0.7457 (0.7405 - 0.7508) | | | |  | Underprediction | 10.7% |
| < 3 visits | 19889 | 217 | 42 | 1 |  |  |  |  |  |  |  | Overprediction | 24.8% |
| 3 - 4 visits | 4728 | 305 | 84 | 12 |  | Class: | < 3 visits | 3 - 4 visits | 5 - 9 visits | ≥ 10 visits |  | Unnecessary predictions | 24.0% |
| 5 - 9 visits | 1350 | 173 | 90 | 15 |  | Sensitivity | 0.7596 | 0.4127 | 0.3502 | 0.3488 |  | Intervention | 75.0% |
| ≥ 10 visits | 216 | 44 | 41 | 15 |  | Specificity | 0.7498 | 0.8179 | 0.9430 | 0.9889 |  | Missed cases | 25.0% |
|  |  |  |  |  |  | PPV | 0.9871 | 0.0595 | 0.0553 | 0.0475 |  |  |  |
|  |  |  |  |  |  | NPV | 0.1101 | 0.9804 | 0.9935 | 0.9990 |  |  |  |
|  |  |  |  |  |  | Prevalence | 0.9618 | 0.0272 | 0.0094 | 0.0016 |  |  |  |
|  |  |  |  |  |  | Detection | 0.7306 | 0.0112 | 0.0033 | 0.0006 |  |  |  |
|  |  |  |  |  |  | Detection | 0.7402 | 0.1884 | 0.0598 | 0.0116 |  |  |  |
|  |  |  |  |  |  | Balanced | 0.7547 | 0.6153 | 0.6466 | 0.6689 |  |  |  |
|  |  |  |  |  |  |  |  |  |  |  |  |  |  |
| **Model: Linear SVM with MARS as the base learner** | | | | | | | | | | | | | |
| Confusion Matrix (test dataset) | | | | |  | Model performance metrics (95% CI) | | | | |  | Additional metrics | |
|  | Reference | | | |  | Multiclass AUC | 0.7722 (0.7316, 0.7681) | |  |  |  | Correct | 30.3% |
| Prediction | < 3 visits | 3 - 4 visits | 5 - 9 visits | ≥ 10 visits |  | Accuracy | 0.7849 (0.78, 0.7898) | |  |  |  | Underprediction | 6.6% |
| < 3 visits | 21052 | 248 | 43 | 2 |  |  |  |  |  |  |  | Overprediction | 34.8% |
| 3 - 4 visits | 3421 | 217 | 54 | 7 |  | Class: | < 3 visits | 3 - 4 visits | 5 - 9 visits | ≥ 10 visits |  | Unnecessary predictions | 19.6% |
| 5 - 9 visits | 1199 | 170 | 72 | 8 |  | Sensitivity | 0.8040 | 0.2936 | 0.2802 | 0.6047 |  | Intervention | 71.8% |
| ≥ 10 visits | 511 | 104 | 88 | 26 |  | Specificity | 0.7180 | 0.8685 | 0.9489 | 0.9741 |  | Missed cases | 28.2% |
|  |  |  |  |  |  | PPV | 0.9863 | 0.0587 | 0.0497 | 0.0357 |  |  |  |
|  |  |  |  |  |  | NPV | 0.1269 | 0.9778 | 0.9928 | 0.9994 |  |  |  |
|  |  |  |  |  |  | Prevalence | 0.9618 | 0.0271 | 0.0094 | 0.0016 |  |  |  |
|  |  |  |  |  |  | Detection | 0.7733 | 0.0080 | 0.0026 | 0.0010 |  |  |  |
|  |  |  |  |  |  | Detection | 0.7841 | 0.1359 | 0.0532 | 0.0268 |  |  |  |
|  |  |  |  |  |  | Balanced | 0.7610 | 0.5811 | 0.6145 | 0.7894 |  |  |  |
|  |  |  |  |  |  |  |  |  |  |  |  |  |  |
| **Model: Random Forest with MLP as the base learner** | | | | | | | | | | | | | |
| **Confusion Matrix (test dataset)** | | | | |  | **Model performance metrics (95% CI)** | | | | |  | **Additional metrics** | |
|  | Reference | | | |  | Multiclass AUC | 0.6248 (0.5110 - 0.5258) | | | |  | Correct | 4.4% |
| Prediction | < 3 visits | 3 - 4 visits | 5 - 9 visits | ≥ 10 visits |  | Accuracy | 0.9553 (0.9527 - 0.9577) | | | |  | Underprediction | 1.7% |
| < 3 visits | 25958 | 725 | 220 | 26 |  |  |  |  |  |  |  | Overprediction | 1.5% |
| 3 - 4 visits | 166 | 20 | 8 | 5 |  | Class: | < 3 visits | 3 - 4 visits | 5 - 9 visits | ≥ 10 visits |  | Unnecessary predictions | 0.8% |
| 5 - 9 visits | 43 | 11 | 12 | 5 |  | Sensitivity | 0.9919 | 0.0264 | 0.0496 | 0.2800 |  | Intervention | 7.6% |
| ≥ 10 visits | 4 | 3 | 2 | 14 |  | Specificity | 0.0761 | 0.9932 | 0.9978 | 0.9997 |  | Missed cases | 92.4% |
|  |  |  |  |  |  | PPV | 0.9639 | 0.1005 | 0.1690 | 0.6087 |  |  |  |
|  |  |  |  |  |  | NPV | 0.2730 | 0.9727 | 0.9915 | 0.9987 |  |  |  |
|  |  |  |  |  |  | Prevalence | 0.9614 | 0.0279 | 0.0089 | 0.0018 |  |  |  |
|  |  |  |  |  |  | Detection | 0.9536 | 0.0007 | 0.0004 | 0.0005 |  |  |  |
|  |  |  |  |  |  | Detection | 0.9892 | 0.0073 | 0.0026 | 0.0008 |  |  |  |
|  |  |  |  |  |  | Balanced | 0.5340 | 0.5098 | 0.5237 | 0.6398 |  |  |  |
|  |  |  |  |  |  |  |  |  |  |  |  |  |  |
| **Model: Random Forest with MLP as the base learner** | | | | | | | | | | | | | |
| **Confusion Matrix (test dataset)** | | | | |  | **Model performance metrics (95% CI)** | | | | |  | **Additional metrics** | |
|  | Reference | | | |  | Multiclass AUC | 0.7557 (0.7310 - 0.7664) | | | |  | Correct | 37.9% |
| Prediction | < 3 visits | 3 - 4 visits | 5 - 9 visits | ≥ 10 visits |  | Accuracy | 0.7432 (0.7380 - 0.7484) | | | |  | Underprediction | 10.4% |
| < 3 visits | 19833 | 226 | 43 | 4 |  |  |  |  |  |  |  | Overprediction | 25.8% |
| 3 - 4 visits | 4713 | 283 | 87 | 11 |  | Class: | < 3 visits | 3 - 4 visits | 5 - 9 visits | ≥ 10 visits |  | Unnecessary predictions | 24.2% |
| 5 - 9 visits | 1446 | 208 | 91 | 11 |  | Sensitivity | 0.7578 | 0.3729 | 0.3760 | 0.4800 |  | Intervention | 74.0% |
| ≥ 10 visits | 179 | 42 | 21 | 24 |  | Specificity | 0.7402 | 0.8182 | 0.9383 | 0.9911 |  | Missed cases | 26.0% |
|  |  |  |  |  |  | PPV | 0.9864 | 0.0556 | 0.0518 | 0.0902 |  |  |  |
|  |  |  |  |  |  | NPV | 0.1093 | 0.9785 | 0.9941 | 0.9990 |  |  |  |
|  |  |  |  |  |  | Prevalence | 0.9614 | 0.0279 | 0.0089 | 0.0018 |  |  |  |
|  |  |  |  |  |  | Detection | 0.7286 | 0.0104 | 0.0033 | 0.0009 |  |  |  |
|  |  |  |  |  |  | Detection | 0.7386 | 0.1871 | 0.0645 | 0.0098 |  |  |  |
|  |  |  |  |  |  | Balanced | 0.7490 | 0.5955 | 0.6572 | 0.7355 |  |  |  |
|  |  |  |  |  |  |  |  |  |  |  |  |  |  |
| **Model: Linear SVM with MLP as the base learner** | | | | | | | | | | | | | |
| **Confusion Matrix (test dataset)** | | | | |  | **Model performance metrics (95% CI)** | | | | |  | **Additional metrics** | |
|  | Reference | | | |  | Multiclass AUC | 0.7959 (0.7164 - 0.7530) | | | |  | Correct | 29.6% |
| Prediction | < 3 visits | 3 - 4 visits | 5 - 9 visits | ≥ 10 visits |  | Accuracy | 0.7883 (0.7834 - 0.7931) | | | |  | Underprediction | 6.5% |
| < 3 visits | 21148 | 279 | 52 | 2 |  |  |  |  |  |  |  | Overprediction | 32.3% |
| 3 - 4 visits | 3127 | 197 | 56 | 3 |  | Class: | < 3 visits | 3 - 4 visits | 5 - 9 visits | ≥ 10 visits |  | Unnecessary predictions | 19.2% |
| 5 - 9 visits | 1655 | 214 | 78 | 9 |  | Sensitivity | 0.8081 | 0.2596 | 0.3223 | 0.7200 |  | Intervention | 68.3% |
| ≥ 10 visits | 241 | 69 | 56 | 36 |  | Specificity | 0.6832 | 0.8796 | 0.9304 | 0.9865 |  | Missed cases | 31.7% |
|  |  |  |  |  |  | PPV | 0.9845 | 0.0582 | 0.0399 | 0.0896 |  |  |  |
|  |  |  |  |  |  | NPV | 0.1251 | 0.9764 | 0.9935 | 0.9995 |  |  |  |
|  |  |  |  |  |  | Prevalence | 0.9614 | 0.0279 | 0.0089 | 0.0018 |  |  |  |
|  |  |  |  |  |  | Detection | 0.7769 | 0.0072 | 0.0029 | 0.0013 |  |  |  |
|  |  |  |  |  |  | Detection | 0.7891 | 0.1243 | 0.0719 | 0.0148 |  |  |  |
|  |  |  |  |  |  | Balanced | 0.7456 | 0.5696 | 0.6264 | 0.8533 |  |  |  |
| MARS: Multivariate Adaptive Regression Splines; MLP: Multi-Layered Perceptron; SVM: Support Vector Machines; AUC: Area under the receiver operator curve; PPV: Positive predictive value; NPV: Negative predictive value. | | | | | | | | | | | | | |

**Table S7**. Detailed model performance metrics for top 5 selected models predicting inpatient bed days (validation dataset).

| **Detailed model performance metrics** | | | | | | | | | | | | | |
| --- | --- | --- | --- | --- | --- | --- | --- | --- | --- | --- | --- | --- | --- |
| **Predicting inpatient bed days category** | | | | | | | | | | | | | |
| **Model: Boosted trees with MARS as base learner** | | | | | | | | | | | | | |
| Confusion Matrix (validation dataset) | | | | |  | Model performance metrics (95% CI) | | | | |  | Additional metrics | |
|  | Reference | | | |  | Multiclass AUC | 0.6811 (0.6986, 0.7140) | |  |  |  | Correct predictions | 30.3% |
| Prediction | <7 days | 7 - 13 days | 14 - 29 days | ≥ 30 days |  | Accuracy | 0.6405 (0.6377, 0.6434) | |  |  |  | Underpredictions | 21.1% |
| <7 days | 67691 | 1311 | 818 | 457 |  |  |  |  |  |  |  | Overpredictions | 25.6% |
| 7 - 13 days | 16866 | 1074 | 878 | 653 |  | Class: | <7 days | 7 - 13 days | 14 - 29 days | ≥ 30 days |  | Unnecessary predictions | 32.1% |
| 14 - 29 days | 9481 | 1121 | 1054 | 839 |  | Sensitivity | 0.6786 | 0.2502 | 0.2832 | 0.3972 |  | Identified utilizers | 77.0% |
| ≥ 30 days | 5719 | 786 | 972 | 1284 |  | Specificity | 0.7701 | 0.8276 | 0.8934 | 0.9306 |  | Missed cases | 23.0% |
|  |  |  |  |  |  | PPV | 0.9632 | 0.0552 | 0.0844 | 0.1466 |  |  |  |
|  |  |  |  |  |  | NPV | 0.2127 | 0.9648 | 0.9729 | 0.9809 |  |  |  |
|  |  |  |  |  |  | Prevalence | 0.8987 | 0.0387 | 0.0335 | 0.0291 |  |  |  |
|  |  |  |  |  |  | Detection Rate | 0.6098 | 0.0097 | 0.0095 | 0.0116 |  |  |  |
|  |  |  |  |  |  | Detection Prevalence | 0.6331 | 0.1754 | 0.1126 | 0.0789 |  |  |  |
|  |  |  |  |  |  | Balanced Accuracy | 0.7243 | 0.5389 | 0.5883 | 0.6639 |  |  |  |
|  |  |  |  |  |  |  |  |  |  |  |  |  |  |
| **Model: Boosted trees with MLP as base learner** | | | | | | | | | | | | | |
| Confusion Matrix (validation dataset) | | | | |  | Model performance metrics (95% CI) | | | | |  | Additional metrics | |
|  | Reference | | | |  | Multiclass AUC | 0.6782 (0.6957, 0.7111) | |  |  |  | Correct predictions | 29.2% |
| Prediction | <7 days | 7 - 13 days | 14 - 29 days | ≥ 30 days |  | Accuracy | 0.6558 (0.6530, 0.6586) | |  |  |  | Underpredictions | 19.2% |
| <7 days | 69513 | 1378 | 855 | 487 |  |  |  |  |  |  |  | Overpredictions | 27.4% |
| 7 - 13 days | 12201 | 852 | 688 | 502 |  | Class: | <7 days | 7 - 13 days | 14 - 29 days | ≥ 30 days |  | Unnecessary predictions | 30.3% |
| 14 - 29 days | 12309 | 1212 | 1158 | 972 |  | Sensitivity | 0.6968 | 0.1985 | 0.3111 | 0.3934 |  | Identified utilizers | 75.8% |
| ≥ 30 days | 5734 | 850 | 1021 | 1272 |  | Specificity | 0.7582 | 0.8745 | 0.8649 | 0.9294 |  | Missed cases | 24.2% |
|  |  |  |  |  |  | PPV | 0.9623 | 0.0598 | 0.0740 | 0.1433 |  |  |  |
|  |  |  |  |  |  | NPV | 0.2199 | 0.9644 | 0.9731 | 0.9808 |  |  |  |
|  |  |  |  |  |  | Prevalence | 0.8987 | 0.0387 | 0.0335 | 0.0291 |  |  |  |
|  |  |  |  |  |  | Detection Rate | 0.6262 | 0.0077 | 0.0104 | 0.0115 |  |  |  |
|  |  |  |  |  |  | Detection Prevalence | 0.6507 | 0.1283 | 0.1410 | 0.0800 |  |  |  |
|  |  |  |  |  |  | Balanced Accuracy | 0.7275 | 0.5365 | 0.5880 | 0.6614 |  |  |  |
|  |  |  |  |  |  |  |  |  |  |  |  |  |  |
| **Model: Boosted trees with logistic regression as base learner** | | | | | | | | | | | | | |
| Confusion Matrix (validation dataset) | | | | |  | Model performance metrics (95% CI) | | | | |  | Additional metrics | |
|  | Reference | | | |  | Multiclass AUC | 0.6767 (0.6929, 0.7082) | |  |  |  | Correct predictions | 29.4% |
| Prediction | <7 days | 7 - 13 days | 14 - 29 days | ≥ 30 days |  | Accuracy | 0.6357 (0.6329, 0.6385) | |  |  |  | Underpredictions | 20.2% |
| <7 days | 67264 | 1313 | 791 | 467 |  |  |  |  |  |  |  | Overpredictions | 27.6% |
| 7 - 13 days | 13474 | 925 | 809 | 517 |  | Class: | <7 days | 7 - 13 days | 14 - 29 days | ≥ 30 days |  | Unnecessary predictions | 32.6% |
| 14 - 29 days | 12774 | 1173 | 1072 | 945 |  | Sensitivity | 0.6743 | 0.2155 | 0.2880 | 0.4033 |  | Identified utilizers | 77.1% |
| ≥ 30 days | 6245 | 881 | 1050 | 1304 |  | Specificity | 0.7714 | 0.8613 | 0.8612 | 0.9241 |  | Missed cases | 22.9% |
|  |  |  |  |  |  | PPV | 0.9632 | 0.0588 | 0.0672 | 0.1376 |  |  |  |
|  |  |  |  |  |  | NPV | 0.2107 | 0.9647 | 0.9721 | 0.9810 |  |  |  |
|  |  |  |  |  |  | Prevalence | 0.8987 | 0.0387 | 0.0335 | 0.0291 |  |  |  |
|  |  |  |  |  |  | Detection Rate | 0.6060 | 0.0083 | 0.0097 | 0.0118 |  |  |  |
|  |  |  |  |  |  | Detection Prevalence | 0.6291 | 0.1417 | 0.1438 | 0.0854 |  |  |  |
|  |  |  |  |  |  | Balanced Accuracy | 0.7228 | 0.5384 | 0.5746 | 0.6637 |  |  |  |
|  |  |  |  |  |  |  |  |  |  |  |  |  |  |
| **Model: Boosted trees with boosted trees as base learner** | | | | | | | | | | | | | |
| Confusion Matrix (validation dataset) | | | | |  | Model performance metrics (95% CI) | | | | |  | Additional metrics | |
|  | Reference | | | |  | Multiclass AUC | 0.6828 (0.6966, 0.7120) | |  |  |  | Correct predictions | 29.9% |
| Prediction | <7 days | 7 - 13 days | 14 - 29 days | ≥ 30 days |  | Accuracy | 0.6496 (0.6468, 0.6524) | |  |  |  | Underpredictions | 21.9% |
| <7 days | 68753 | 1347 | 844 | 458 |  |  |  |  |  |  |  | Overpredictions | 24.7% |
| 7 - 13 days | 16058 | 1120 | 938 | 646 |  | Class: | <7 days | 7 - 13 days | 14 - 29 days | ≥ 30 days |  | Unnecessary predictions | 31.1% |
| 14 - 29 days | 9602 | 1062 | 984 | 874 |  | Sensitivity | 0.6892 | 0.2610 | 0.2644 | 0.3882 |  | Identified utilizers | 76.4% |
| ≥ 30 days | 5344 | 763 | 956 | 1255 |  | Specificity | 0.7645 | 0.8347 | 0.8925 | 0.9345 |  | Missed cases | 23.6% |
|  |  |  |  |  |  | PPV | 0.9629 | 0.0597 | 0.0786 | 0.1509 |  |  |  |
|  |  |  |  |  |  | NPV | 0.2171 | 0.9656 | 0.9722 | 0.9807 |  |  |  |
|  |  |  |  |  |  | Prevalence | 0.8987 | 0.0387 | 0.0335 | 0.0291 |  |  |  |
|  |  |  |  |  |  | Detection Rate | 0.6194 | 0.0101 | 0.0089 | 0.0113 |  |  |  |
|  |  |  |  |  |  | Detection Prevalence | 0.6432 | 0.1690 | 0.1128 | 0.0749 |  |  |  |
|  |  |  |  |  |  | Balanced Accuracy | 0.7268 | 0.5478 | 0.5784 | 0.6613 |  |  |  |
|  |  |  |  |  |  |  |  |  |  |  |  |  |  |
| **Model: Linear SVM with MLP as base learner** | | | | | | | | | | | | | |
| Confusion Matrix (validation dataset) | | | | |  | Model performance metrics (95% CI) | | | | |  | Additional metrics | |
|  | Reference | | | |  | Multiclass AUC | 0.6742 (0.6851, 0.7004) | |  |  |  | Correct predictions | 26.5% |
| Prediction | <7 days | 7 - 13 days | 14 - 29 days | ≥ 30 days |  | Accuracy | 0.7100 (0.7073, 0.7127) | |  |  |  | Underpredictions | 19.3% |
| <7 days | 75830 | 1668 | 1069 | 595 |  |  |  |  |  |  |  | Overpredictions | 24.5% |
| 7 - 13 days | 5883 | 393 | 308 | 265 |  | Class: | <7 days | 7 - 13 days | 14 - 29 days | ≥ 30 days |  | Unnecessary predictions | 24.0% |
| 14 - 29 days | 14919 | 1848 | 1818 | 1603 |  | Sensitivity | 0.7601 | 0.0916 | 0.4885 | 0.2382 |  | Identified utilizers | 70.4% |
| ≥ 30 days | 3125 | 383 | 527 | 770 |  | Specificity | 0.7037 | 0.9395 | 0.8288 | 0.9626 |  | Missed cases | 29.6% |
|  |  |  |  |  |  | PPV | 0.9579 | 0.0574 | 0.0901 | 0.1603 |  |  |  |
|  |  |  |  |  |  | NPV | 0.2486 | 0.9626 | 0.9790 | 0.9768 |  |  |  |
|  |  |  |  |  |  | Prevalence | 0.8987 | 0.0387 | 0.0335 | 0.0291 |  |  |  |
|  |  |  |  |  |  | Detection Rate | 0.6831 | 0.0035 | 0.0164 | 0.0069 |  |  |  |
|  |  |  |  |  |  | Detection Prevalence | 0.7131 | 0.0617 | 0.1819 | 0.0433 |  |  |  |
|  |  |  |  |  |  | Balanced Accuracy | 0.7319 | 0.5155 | 0.6586 | 0.6004 |  |  |  |
| MARS: Multivariate Adaptive Regression Splines; MLP: Multi-Layered Perceptron; SVM: Support Vector Machines; AUC: Area under the receiver operator curve; PPV: Positive predictive value; NPV: Negative predictive value. | | | | | | | | | | | | | |

**Table S8**. Detailed model performance metrics for top 5 selected models predicting emergency department visits (validation dataset).

| **Detailed model performance metrics** | | | | | | | | | | | | | |
| --- | --- | --- | --- | --- | --- | --- | --- | --- | --- | --- | --- | --- | --- |
| **Predicting emergency department visits category** | | | | | | | | | | | | | |
| **Model: Boosted trees with MARS as base learner** | | | | | | | | | | | | | |
| Confusion Matrix (validation dataset) | | | | |  | Model performance metrics (95% CI) | | | | |  | Additional metrics | |
|  | Reference | | | |  | Multiclass AUC | 0.7664 (0.7169, 0.7332) | |  |  |  | Correct | 36.7% |
| Prediction | < 3 visits | 3 - 4 visits | 5 - 9 visits | ≥ 10 visits |  | Accuracy | 0.7266 (0.7239, 0.7292) | |  |  |  | Underprediction | 10.2% |
| < 3 visits | 78649 | 1262 | 285 | 9 |  |  |  |  |  |  |  | Overprediction | 24.7% |
| 3 - 4 visits | 20133 | 1394 | 450 | 29 |  | Class: | < 3 visits | 3 - 4 visits | 5 - 9 visits | ≥ 10 visits |  | Unnecessary predictions | 25.5% |
| 5 - 9 visits | 5905 | 962 | 533 | 76 |  | Sensitivity | 0.7452 | 0.3652 | 0.3663 | 0.4000 |  | Intervention | 71.5% |
| ≥ 10 visits | 855 | 199 | 187 | 76 |  | Specificity | 0.7151 | 0.8077 | 0.9366 | 0.9888 |  | Missed cases | 28.5% |
|  |  |  |  |  |  | PPV | 0.9806 | 0.0634 | 0.0713 | 0.0577 |  |  |  |
|  |  |  |  |  |  | NPV | 0.1268 | 0.9728 | 0.9911 | 0.9990 |  |  |  |
|  |  |  |  |  |  | Prevalence | 0.9508 | 0.0344 | 0.0131 | 0.0017 |  |  |  |
|  |  |  |  |  |  | Detection Rate | 0.7085 | 0.0126 | 0.0048 | 0.0007 |  |  |  |
|  |  |  |  |  |  | Detection Prevalence | 0.7225 | 0.1983 | 0.0673 | 0.0119 |  |  |  |
|  |  |  |  |  |  | Balanced Accuracy | 0.7302 | 0.5865 | 0.6515 | 0.6944 |  |  |  |
|  |  |  |  |  |  |  |  |  |  |  |  |  |  |
| **Model: Boosted trees with logistic regression as base learner** | | | | | | | | | | | | | |
| Confusion Matrix (validation dataset) | | | | |  | Model performance metrics (95% CI) | | | | |  | Additional metrics | |
|  | Reference | | | |  | Multiclass AUC | 0.7669 (0.7199, 0.7359) | |  |  |  | Correct | 39.8% |
| Prediction | < 3 visits | 3 - 4 visits | 5 - 9 visits | ≥ 10 visits |  | Accuracy | 0.7053 (0.7026, 0.7079) | |  |  |  | Underprediction | 10.4% |
| < 3 visits | 76114 | 1155 | 260 | 10 |  |  |  |  |  |  |  | Overprediction | 23.7% |
| 3 - 4 visits | 22660 | 1554 | 468 | 31 |  | Class: | < 3 visits | 3 - 4 visits | 5 - 9 visits | ≥ 10 visits |  | Unnecessary predictions | 27.9% |
| 5 - 9 visits | 6102 | 939 | 538 | 69 |  | Sensitivity | 0.7212 | 0.4071 | 0.3698 | 0.4211 |  | Intervention | 73.9% |
| ≥ 10 visits | 666 | 169 | 189 | 80 |  | Specificity | 0.7391 | 0.7839 | 0.9351 | 0.9908 |  | Missed cases | 26.1% |
|  |  |  |  |  |  | PPV | 0.9816 | 0.0629 | 0.0703 | 0.0725 |  |  |  |
|  |  |  |  |  |  | NPV | 0.1206 | 0.9738 | 0.9911 | 0.9990 |  |  |  |
|  |  |  |  |  |  | Prevalence | 0.9508 | 0.0344 | 0.0131 | 0.0017 |  |  |  |
|  |  |  |  |  |  | Detection Rate | 0.6857 | 0.0140 | 0.0048 | 0.0007 |  |  |  |
|  |  |  |  |  |  | Detection Prevalence | 0.6985 | 0.2226 | 0.0689 | 0.0099 |  |  |  |
|  |  |  |  |  |  | Balanced Accuracy | 0.7301 | 0.5955 | 0.6524 | 0.7059 |  |  |  |
|  |  |  |  |  |  |  |  |  |  |  |  |  |  |
| **Model: Boosted trees with MLP as base learner** | | | | | | | | | | | | | |
| Confusion Matrix (validation dataset) | | | | |  | Model performance metrics (95% CI) | | | | |  | Additional metrics | |
|  | Reference | | | |  | Multiclass AUC | 0.7563 (0.7158, 0.7320) | |  |  |  | Correct | 36.9% |
| Prediction | < 3 visits | 3 - 4 visits | 5 - 9 visits | ≥ 10 visits |  | Accuracy | 0.7269 (0.7243, 0.7295) | |  |  |  | Underprediction | 10.5% |
| < 3 visits | 78675 | 1264 | 296 | 9 |  |  |  |  |  |  |  | Overprediction | 23.9% |
| 3 - 4 visits | 19904 | 1401 | 459 | 38 |  | Class: | < 3 visits | 3 - 4 visits | 5 - 9 visits | ≥ 10 visits |  | Unnecessary predictions | 25.5% |
| 5 - 9 visits | 6165 | 975 | 547 | 78 |  | Sensitivity | 0.7454 | 0.3670 | 0.3759 | 0.3421 |  | Intervention | 71.3% |
| ≥ 10 visits | 798 | 177 | 153 | 65 |  | Specificity | 0.7127 | 0.8097 | 0.9341 | 0.9898 |  | Missed cases | 28.7% |
|  |  |  |  |  |  | PPV | 0.9804 | 0.0643 | 0.0704 | 0.0545 |  |  |  |
|  |  |  |  |  |  | NPV | 0.1266 | 0.9729 | 0.9912 | 0.9989 |  |  |  |
|  |  |  |  |  |  | Prevalence | 0.9508 | 0.0344 | 0.0131 | 0.0017 |  |  |  |
|  |  |  |  |  |  | Detection Rate | 0.7088 | 0.0126 | 0.0049 | 0.0006 |  |  |  |
|  |  |  |  |  |  | Detection Prevalence | 0.7229 | 0.1964 | 0.0700 | 0.0107 |  |  |  |
|  |  |  |  |  |  | Balanced Accuracy | 0.7291 | 0.5884 | 0.6550 | 0.6660 |  |  |  |
|  |  |  |  |  |  |  |  |  |  |  |  |  |  |
| **Model: Boosted trees with boosted trees as base learner** | | | | | | | | | | | | | |
| Confusion Matrix (validation dataset) | | | | |  | Model performance metrics (95% CI) | | | | |  | Additional metrics | |
|  | Reference | | | |  | Multiclass AUC | 0.7577 (0.7139, 0.7300) | |  |  |  | Correct | 39.7% |
| Prediction | < 3 visits | 3 - 4 visits | 5 - 9 visits | ≥ 10 visits |  | Accuracy | 0.7317 (0.7290, 0.7343) | |  |  |  | Underprediction | 12.5% |
| < 3 visits | 79050 | 1279 | 285 | 12 |  |  |  |  |  |  |  | Overprediction | 19.0% |
| 3 - 4 visits | 21608 | 1617 | 568 | 44 |  | Class: | < 3 visits | 3 - 4 visits | 5 - 9 visits | ≥ 10 visits |  | Unnecessary predictions | 25.1% |
| 5 - 9 visits | 4313 | 800 | 486 | 70 |  | Sensitivity | 0.7490 | 0.4236 | 0.3340 | 0.3368 |  | Intervention | 71.1% |
| ≥ 10 visits | 571 | 121 | 116 | 64 |  | Specificity | 0.7115 | 0.7927 | 0.9527 | 0.9927 |  | Missed cases | 28.9% |
|  |  |  |  |  |  | PPV | 0.9805 | 0.0678 | 0.0857 | 0.0734 |  |  |  |
|  |  |  |  |  |  | NPV | 0.1279 | 0.9748 | 0.9908 | 0.9989 |  |  |  |
|  |  |  |  |  |  | Prevalence | 0.9508 | 0.0344 | 0.0131 | 0.0017 |  |  |  |
|  |  |  |  |  |  | Detection Rate | 0.7121 | 0.0146 | 0.0044 | 0.0006 |  |  |  |
|  |  |  |  |  |  | Detection Prevalence | 0.7263 | 0.2147 | 0.0511 | 0.0079 |  |  |  |
|  |  |  |  |  |  | Balanced Accuracy | 0.7302 | 0.6082 | 0.6434 | 0.6648 |  |  |  |
|  |  |  |  |  |  |  |  |  |  |  |  |  |  |
| **Model: Linear SVM with MARS as base learner** | | | | | | | | | | | | | |
| Confusion Matrix (validation dataset) | | | | |  | Model performance metrics (95% CI) | | | | |  | Additional metrics | |
|  | Reference | | | |  | Multiclass AUC | 0.7818 (0.7204, 0.7368) | |  |  |  | Correct | 30.1% |
| Prediction | < 3 visits | 3 - 4 visits | 5 - 9 visits | ≥ 10 visits |  | Accuracy | 0.7656 (0.7631, 0.7681) | |  |  |  | Underprediction | 6.7% |
| < 3 visits | 83339 | 1384 | 300 | 9 |  |  |  |  |  |  |  | Overprediction | 32.2% |
| 3 - 4 visits | 14515 | 1099 | 312 | 14 |  | Class: | < 3 visits | 3 - 4 visits | 5 - 9 visits | ≥ 10 visits |  | Unnecessary predictions | 21.0% |
| 5 - 9 visits | 5682 | 823 | 417 | 38 |  | Sensitivity | 0.7896 | 0.2879 | 0.2866 | 0.6789 |  | Intervention | 69.0% |
| ≥ 10 visits | 2006 | 511 | 426 | 129 |  | Specificity | 0.6900 | 0.8615 | 0.9403 | 0.9734 |  | Missed cases | 31.0% |
|  |  |  |  |  |  | PPV | 0.9801 | 0.0689 | 0.0599 | 0.0420 |  |  |  |
|  |  |  |  |  |  | NPV | 0.1451 | 0.9714 | 0.9900 | 0.9994 |  |  |  |
|  |  |  |  |  |  | Prevalence | 0.9508 | 0.0344 | 0.0131 | 0.0017 |  |  |  |
|  |  |  |  |  |  | Detection Rate | 0.7508 | 0.0099 | 0.0038 | 0.0012 |  |  |  |
|  |  |  |  |  |  | Detection Prevalence | 0.7660 | 0.1436 | 0.0627 | 0.0277 |  |  |  |
|  |  |  |  |  |  | Balanced Accuracy | 0.7398 | 0.5747 | 0.6134 | 0.8262 |  |  |  |
| MARS: Multivariate Adaptive Regression Splines; MLP: Multi-Layered Perceptron; SVM: Support Vector Machines; AUC: Area under the receiver operator curve; PPV: Positive predictive value; NPV: Negative predictive value. | | | | | | | | | | | | | |

**Figure S1**. Two-axis plot for the distribution of gross inpatient bill (per calendar year) in the SDR.

***
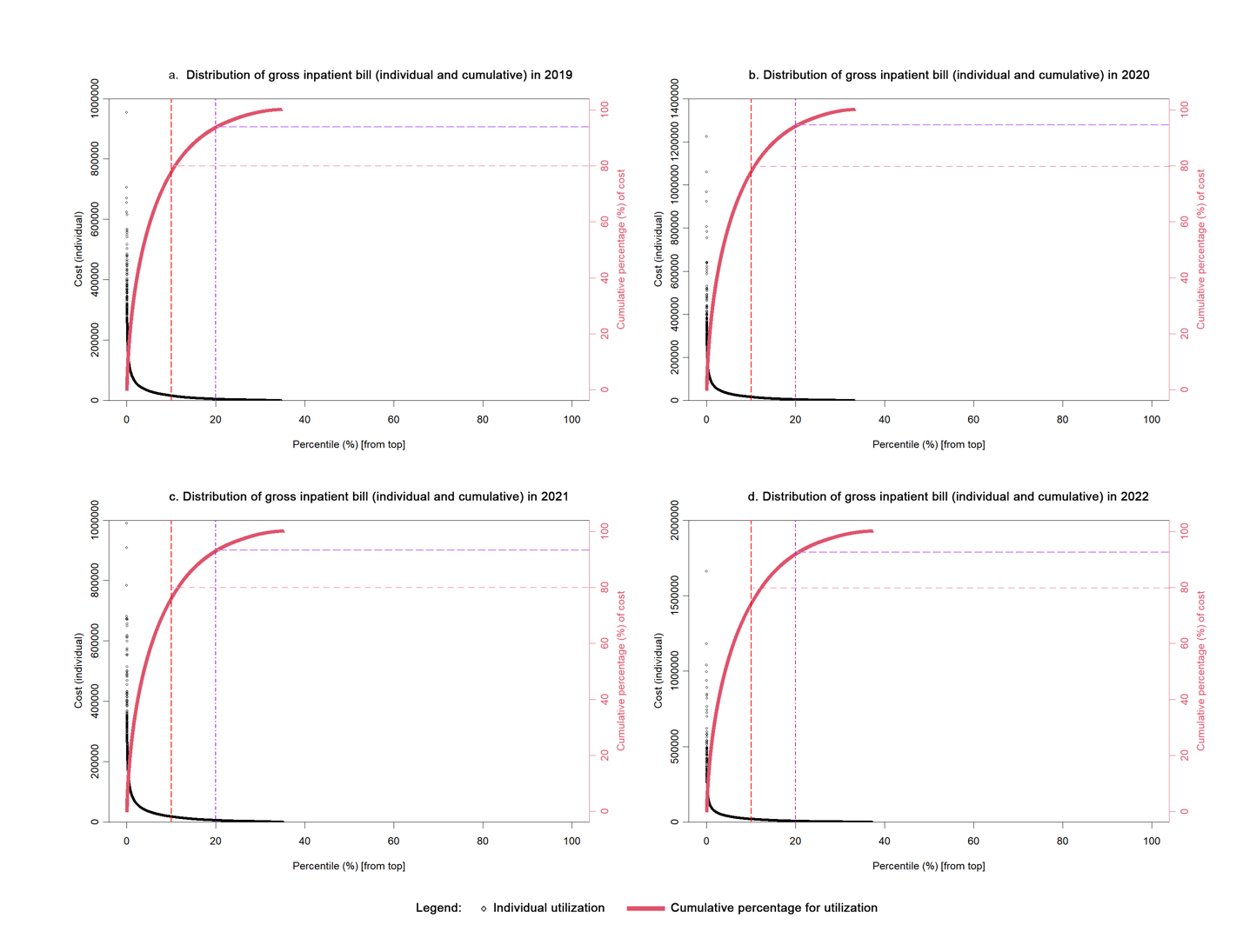
***

**Figure S2.** Two-axis plot for the distribution of total inpatient bed days in the SDR.**
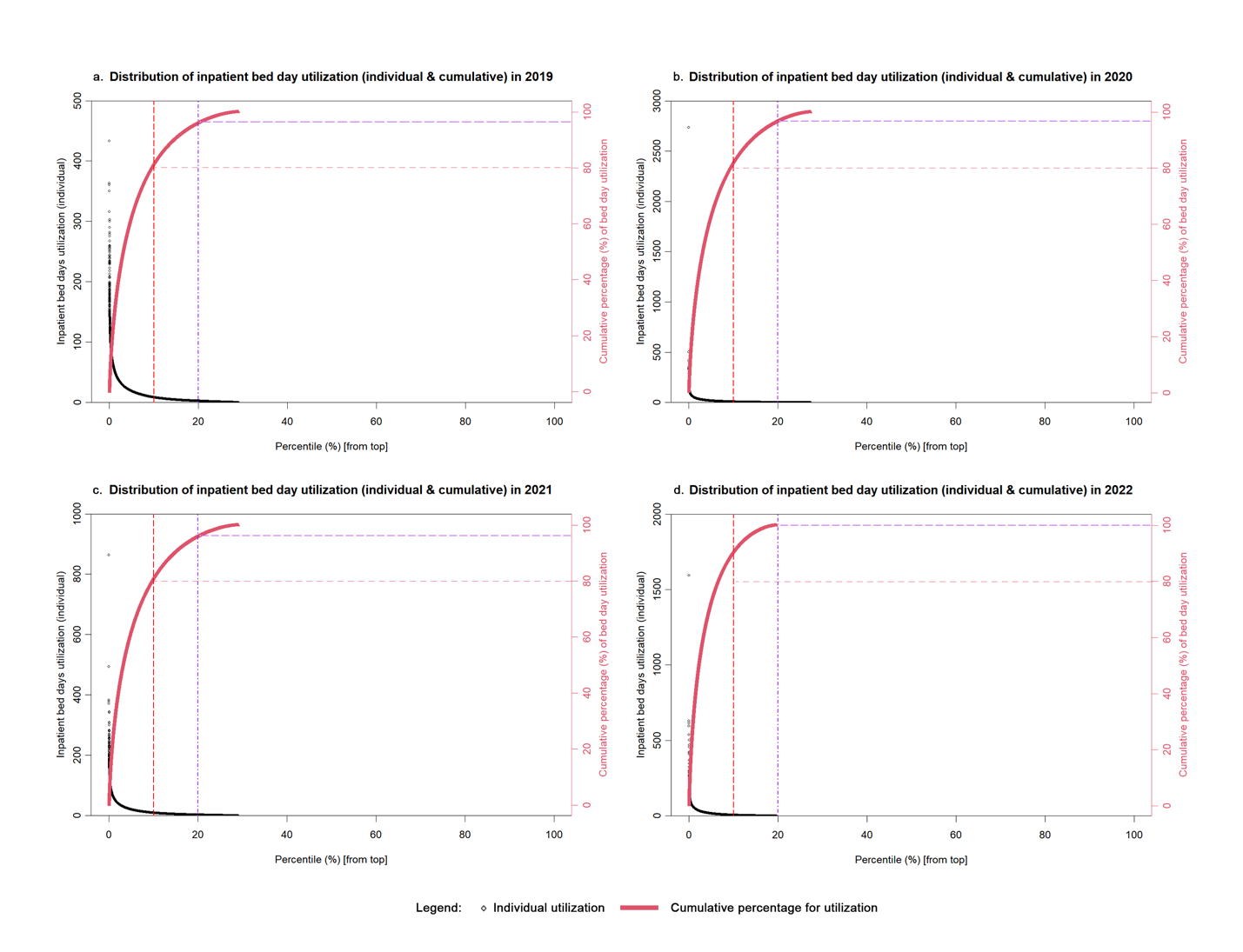
**

**Figure S3.** Scatterplots demonstrating the relationship between gross inpatient bill and inpatient bed days.
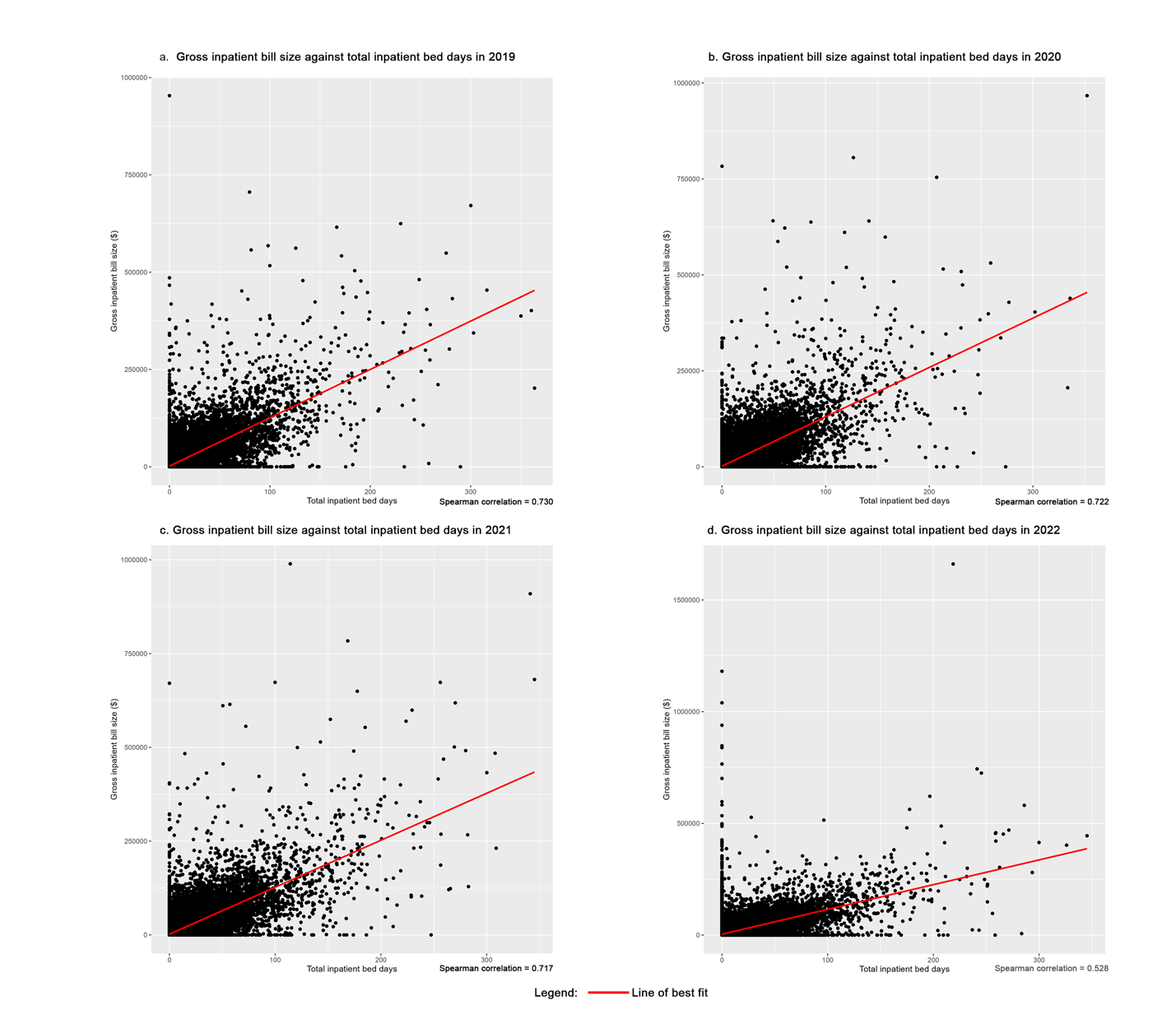


**Figure S4**. Boxplot for Monte Carlo simulations
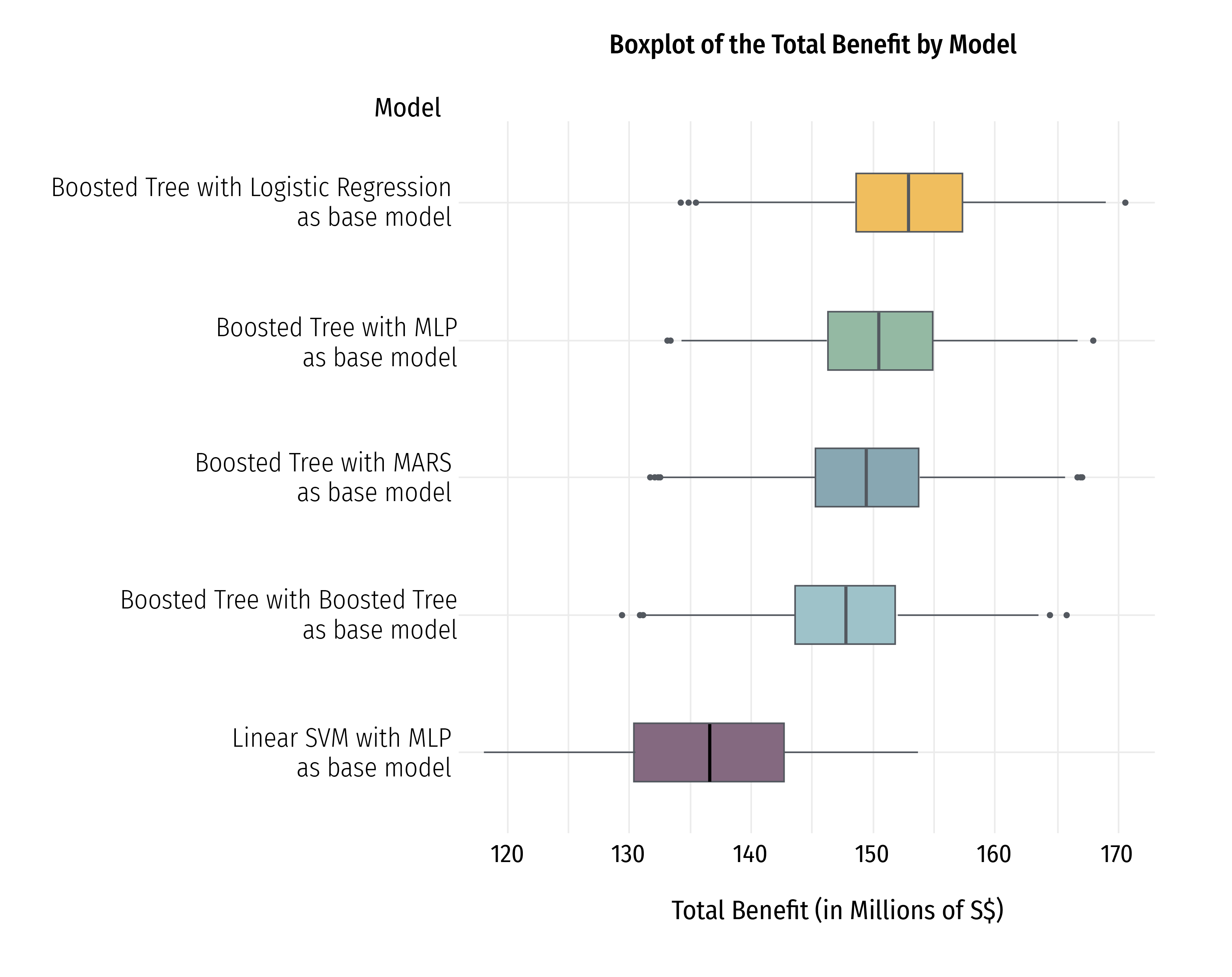

Supplement: Multimedia Appendix 1 [file medinform_v14i1e77202_app1.docx]
